# The metabolite alpha-ketobutyrate extends lifespan by promoting peroxisomal function in *C. elegans*

**DOI:** 10.1038/s41467-023-35899-1

**Published:** 2023-01-16

**Authors:** Nan Wu, Yi-Cheng Ma, Xin-Qian Gong, Pei-Ji Zhao, Yong-Jian Jia, Qiu Zhao, Jia-Hong Duan, Cheng-Gang Zou

**Affiliations:** State key Laboratory for Conservation and Utilization of Bio-Resources in Yunnan, School of Life Sciences, Yunnan University, Kunming, Yunnan 650091 China

**Keywords:** Senescence, Autophagy, Peroxisomes

## Abstract

Metabolism is intimately linked to aging. There is a growing number of studies showing that endogenous metabolites may delay aging and improve healthspan. Through the analysis of existing transcriptome data, we discover a link between activation of the transsulfuration pathway and a transcriptional program involved in peroxisome function and biogenesis in long-lived *glp-1*(*e2141ts*) mutant *Caenorhabditis elegans* worms. Subsequently, we show that supplementation with α-ketobutyrate, an intermediate of the transsulfuration pathway, extends lifespan in wild-type worms. Alpha-ketobutyrate augments the production of NAD^+^ via the lactate dehydrogenase LDH-1, leading to SIR-2.1/SIRT1-mediated enhanced peroxisome function and biogenesis, along with a concomitant increase in the expression of *acox-1.2/ACOX1* in the peroxisomal fatty acid β-oxidation pathway. ACOX-1.2/ACOX1 promotes H_2_O_2_ formation, thereby resulting in activation of SKN-1/NRF2. This transcription factor in turn extends the lifespan of worms by driving expression of autophagic and lysosomal genes. Finally, we show that α-ketobutyrate also delays the cellular senescence in fibroblast cells through the SIRT1-ACOX1-H_2_O_2_-NRF2 pathway. This finding uncovers a previously unknown role for α-ketobutyrate in organismal lifespan and healthspan by coordinating the NAD^+^-SIRT1 signaling and peroxisomal function.

## Abstract (web summary)

Understanding how metabolites modulate longevity is crucial for reducing aging-related disease. Here, the authors demonstrate that α-ketobutyrate exhibits an anti-aging effect by coordinating NAD + -SIRT1 signaling and peroxisome function.

## Introduction

The transsulfuration pathway (TSP) is an evolutionarily conserved pathway that commits methionine to cysteine synthesis in metazoan^1–3^. There are two key enzymes, cystathionine β-synthase (CBS) and cystathionase (CTH, also called cystathionine γ-lyase, GCL), in this pathway. CBS converts serine and homocysteine into cystathionine, while CTH catalyzes the elimination of cystathionine to form cysteine and α-ketobutyrate (α-KB)^1,2^. Both CBS and CTH catalyze the production of a gas hydrogen sulfide (H_2_S) via condensation of cysteine and homocysteine, and desulfhydration of cysteine, respectively^2,4^. In the fruit fly *Drosophila melanogaster* and the nematode *Caenorhabditis elegans*, overexpression of CBS extends lifespan, which is probably due to increased H_2_S production^4,5^. As a product of TSP, H_2_S can function as a signaling molecule to extend lifespan and improve healthspan^4,6,7^. Although the underlying mechanism remains unknown, supplementation with NaHS, a compound that can produce H_2_S, increases the NAD^+^ levels in mammalian cells^8^. It has been well-established that elevation of intracellular NAD^+^ levels by supplementation of NAD^+^ precursors such as nicotinamide mononucleotide (NAM) and nicotinamide mononucleotide (NMN), or activation of the de novo NAD^+^ synthesis pathway, promotes healthspan and extends lifespan via a mechanism requiring SIRT1/SIR-2.1 across species from worms to mammals^9–12^. However, the role of α-KB, another product of TSP, in the regulation of longevity remains unknown.

In *C. elegans*, *glp-1* encodes a Notch receptor crucial for the regulation of the germline stem cell pool^13^. The germline can be removed by blocking germline stem-cell proliferation with the temperature-sensitive (ts) *glp-1(e2144ts)* mutation^14,15^. The germ-cell loss extends *C. elegans* lifespan through activating transcription factors such as DAF-16/FOXO and SKN-1/NRF2 and nuclear hormone receptors such as DAF-12 and NHR-80^14–22^. Interestingly, the levels of H_2_S production are also elevated, which is mediated by reactive oxygen species (ROS) derived from mitochondria, in the germline-deficient *glp-1*(*e2141ts*) mutants. H_2_S promotes the nuclear translocation of SKN-1, thereby extending lifespan in the germline-deficient worms^15^. Besides *glp-1*(*e2141ts*) mutants, ROS levels are elevated in respiration mutants, such as *clk-1*(*qm30*), *isp-1*(*qm150*), and *nuo-6*(*qm200*) mutants, which are responsible for longevity extension by increasing the activity of hypoxia-inducible factor HIF-1^23,24^. Meanwhile, lifespan is extended in response to increased levels of ROS through treatment with 2-deoxyglucose or a low dosage of the oxidant paraquat in worms^25–27^. These data suggest that ROS act as signaling molecules to regulate lifespan.

By analyzing existing transcriptome data of long-lived *glp-1(e2141ts)* mutants in two independent studies (GSE43864 and GSE63075)^20,28^, we observed an upregulation of a set of peroxisome-related genes, which are involved in peroxisomal biogenesis, peroxisomal fatty acid β-oxidation, and amino acid metabolism. These data implicate a role of enhanced peroxisomal function in lifespan extension in germline-deficient worms. As a representative of highly plastic organelles, peroxisomes are capable of modifying their size, abundance, and morphology in response to cellular or environmental cues^29^. The peroxisome has been implicated in aging and longevity regulation^30–33^, yet the exact role and mechanism of action of peroxisome in longevity are far from fully elucidated.

In this study, we demonstrated that the levels of α-KB, another product of TSP, were elevated in germline-deficient *glp-1*(*e2141ts*) mutants. Supplementation with α-KB promoted peroxisome biogenesis, leading to an increase in H_2_O_2_ production, in an NAD^+^-SIRT1 signal-dependent manner. H_2_O_2_, in turn, induced autophagy via SKN-1/NRF2, thereby extending lifespan in the germline-deficient animals. Finally, our data demonstrated that α-KB treatment reduced cellular senescence in mammalian cells. These findings provide novel pharmacological approaches to prevent age-related physiological decline.

## Results

### Alpha-KB extends the lifespan in worms

**Fig. 1:**
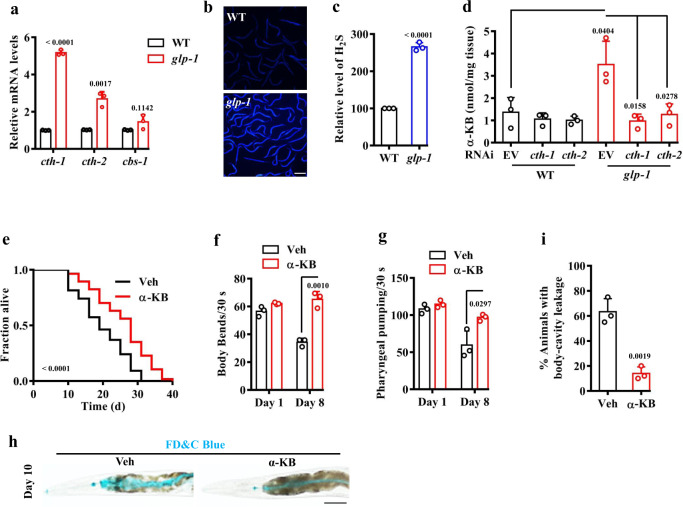
Alpha-KB promotes lifespan in worms. **a** The mRNA levels of *cth-1* and *cth-2*, but not *cbs-1*, were upregulated in *glp-1*(*e2141ts*) mutants, compared with those in wild-type (WT) worms. These results are means ± SD of three independent experiments. **b**, **c** Representative images of H_2_S formation detected by a fluorescent probe. A similar pattern of H2S formation was observed in three independent experiments. Scale bars: 500 μm. **c** Quantification of fluorescent intensity in **b**. The content of H_2_S was increased in *glp-1(e2141ts)* mutants, compared with that in WT worms. Data were presented as mean values ± SEM of three independent experiments (*n* = 35 worms per experiment). *P* values (**a**, **c**) were calculated using the two-sample *t*-test. **d** The levels of α-ketobutyrate (α-KB) were increased in *glp-1(e2141ts)* mutants, compared with that in WT worms. RNAi knockdown of either *cth-1* or *cth-2* reduced the contents of α-KB in *glp-1*(*e2141ts*) mutants back to those of WT worms. Data were presented as mean values ± SEM of three independent experiments. **e** Supplementation with α-KB (500 μM) extended lifespan in WT worms. Veh vehicle. *P* value was calculated using a log-rank test. See survival statistics in Supplementary Data 1. **f**–**i** Supplementation with α-KB delayed the appearance of the aging markers, including pharyngeal pumping (**f**), body bending (**g**), and body-cavity leakages (**h**, **i**) in WT worms. Scale bars: 500 μm. Data were presented as mean values ± SEM of three independent experiments (*n* = 25 worms per experiment). *P* values (**d**, **f**, **g**, **i**) were calculated using a one-way ANOVA followed by a Student–Newman–Keuls test. Source data are provided as a Source Data file.

We analyzed the transcriptome data of *glp-1*(*e2141ts*) mutants in two independent studies^20,28^, and identified that there were 3208 overlapped genes upregulated between the two datasets (Supplementary Fig. 1a), including the major genes in TSP, such as *cth-1*, *cth-2*, and *cbs-1* (Supplementary Fig. 1b). These results implicate that TSP is activated in germline-deficient worms. To assess whether TSP is activated in germline-deficient *glp-1*(*e2141ts*) mutants, we first determined the expressions of *cbs-1*, *cth-1*, and *cth-2*, the major genes in TSP^4,7^. Using qPCR, we found that the expressions of *cth-1*, and *cth-2*, but not *cbs-1*, were markedly upregulated in germline-deficient animals (Fig. 1a). Previously, we developed a fluorescent chemodosimeter for monitoring endogenous H_2_S in mammalian cells, flies and worms^34^. Using the fluorescent probe, we found that the relative levels of H_2_S were significantly increased in germline-deficient animals (Fig. 1b, c), which was consistent with a previous study^15^. These data suggest that TSP is activated in germline-deficient worms. We then asked whether another product of TSP, α-KB, which is derived from the elimination of cystathionine catalyzed by CTH^2,3^, was elevated in these animals. Using LC-MS, we found that the levels of α-KB were significantly increased in germline-deficient mutants, compared with those in wild-type (WT) worms (Fig. 1d). RNAi knockdown of either *cth-1* or *cth-2* reduced the levels of α-KB in germline-deficient worms back to those of WT worms (Fig. 1d).

A previous study has demonstrated that the knockdown of *cth-2* does not affect the lifespan of WT animals^35^. Likewise, the *cth-1(ok3319)* mutants do not exhibit an overall significantly shorter maximal lifespan although these animals initially display a much higher death rate^36^. Consistent with these observations, we found that RNAi knockdown of either *cth-1* or *cth-2* did not alter the lifespan of WT worms (Supplementary Fig. 2a, b and Supplementary Data 1). However, RNAi knockdown of either *cth-1* or *cth-2* shortened the lifespan of germline-deficient worms (Supplementary Fig. 2a, b). Although supplementation with α-KB did not further extend the lifespan of germline-deficient worms, it partially restored the lifespan of germline-deficient worms subjected to *cth-1* or *cth-2* RNAi (Supplementary Fig. 2a, b). In addition, we determined the effect of α-KB supplementation on lifespan in other three longevity pathways: DR (the feeding deficient mutant *eat-2*), reduced insulin/IGF-1 signaling (the receptor mutant *daf-2*), reduced mitochondrial respiration (the mitochondrial complex III mutant *isp-1*). We found that supplementation with α-KB could extend the lifespan of *eat-2(ad1116)*, but not *isp-1(qm150)* and *daf-2(e1370)* (Supplementary Fig. 3a–c and Supplementary Data 1).

Considering both CTH-1 and CTH-2 are H_2_S-producing enzymes^1,2^, we tested the combined effects of α-KB and H_2_S. Like α-KB, supplementation with NaHS (300 μM) also partially restored the lifespan of *glp-1(e2141ts)* mutants subjected to either *cth-1* or *cth-2* RNAi (Supplementary Fig. 2a, b). However, supplementation with both α-KB and NaHS did fully restore the lifespan of *glp-1(e2141ts)* mutants subjected to either *cth-1* or *cth-2* RNAi (Supplementary Fig. 2a, b). These results indicate that the upregulation of either *cth-1* or *cth-2* contributes to lifespan extension in *glp-1(e2141ts)* mutants via both α-KB and H_2_S.

Next, we investigated the effect of α-KB in WT worms and found that supplementation with α-KB significantly extended the lifespan of WT worms (Fig. 1e and Supplementary Data 1). Moreover, we determined the effect of α-KB on several age-associated markers, pharyngeal-pumping rate, body bending, lipofuscin autofluorescence^37,38^, the integrity of intestinal barrier, and aggregation of polyglutamine::YFP (Poly-Q35::YFP)^39^. As expected, the reduction in both pharyngeal-pumping rate (Fig. 1f) and body bending (Fig. 1g) in 8-day-old worms was significantly inhibited after α-KB treatment. We detected the integrity of the intestinal barrier using food dye FD&C Blue No. 1, and found that the body-cavity leakage in 10-day-old worms treated with α-KB than that in age-matched worms with vehicle (Fig. 1h, i). In contrast, supplementation with α-KB did not affect the number of Poly-Q35::YFP aggregates in body wall muscle cells of 8-day-old worms (Supplementary Fig. 4a, b). Taken together, these results suggest that α-KB treatment delays the aging process in worms.

### Lifespan extension involves NAD^+^ production in a SIR-2.1-dependent fashion

**Fig. 2:**
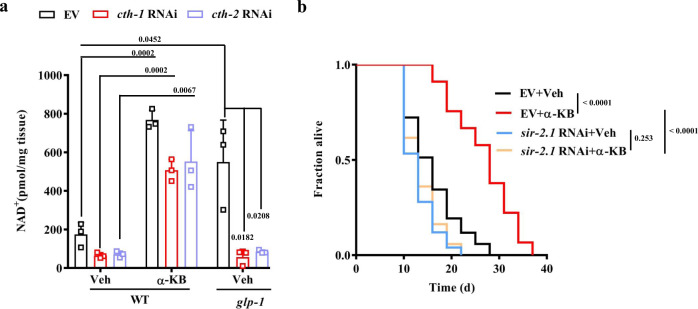
Lifespan extension by α-KB depends on the NAD^+^-SIR-2.1 pathway. **a** The contents of NAD^+^ were increased in α-ketobutyrate (α-KB, 500 μM) -treated wild-type (WT) worms or *glp-1*(*e2141ts*) mutants. Knockdown of either *cth-1* or *cth-2* by RNAi reduced the levels of NAD^+^ in germline-deficient worms back to those of WT worms. Veh vehicle. Data were presented as mean values ± SEM of three independent experiments. *P* value was calculated using a one-way ANOVA followed by a Student–Newman–Keuls test. **b** RNAi knockdown of *sir-2.1* inhibited lifespan extension by α-KB (500 μM) in WT worms. *P* values were calculated using a log-rank test. See survival statistics in Supplementary Data 1. Source data are provided as a Source Data file.

Increased oxidized nicotinamide adenine dinucleotide (NAD^+^) levels improve lifespan and healthspan across species, including worms^10,11,40–42^. Alpha-KB is readily reduced via lactate dehydrogenase or other intracellular dehydrogenases to generate NAD^+^^43,44^. Indeed, using LC-MS, we found that supplementation with α-KB increased the NAD^+^ levels in WT worms (Fig. 2a). Knockdown of either *cth-1* or *cth-2* by RNAi reduced the levels of NAD^+^ in WT worms, which were restored by supplementation with α-KB. Furthermore, an increase in the NAD^+^ levels was observed in *glp-1*(*e2141ts*) mutants (Fig. 2a). Knockdown of either *cth-1* or *cth-2* by RNAi reduced the levels of NAD^+^ in germline-deficient worms back to those of WT worms (Fig. 2a).

The beneficial effects of NAD^+^ on lifespan extension depend on SIR-2.1 (the *C. elegans* ortholog of SIRT1), a member of the sirtuin family of NAD^+^-dependent deacylases^10,11,41,42^. We then asked whether SIR-2.1 was involved in α-KB-mediated lifespan extension. RNAi knockdown of *sir-2.1* completely blocked lifespan extension by α-KB treatment (Fig. 2b and Supplementary Data 1) but only partially reduced lifespan extension by the *glp-1(e2141ts)* mutation (Supplementary Fig. 5 and Supplementary Data 1). Thus, SIR-2.1 is specifically required for α-KB-mediated lifespan extension. By contrast, SIR-2.1 is not specific to longevity in *glp-1(e2141ts)* mutants. Taken together, these results suggest that α-KB extends the lifespan by increasing NAD^+^ levels and its effect depends on the concomitant activity of SIR-2.1.

### Alpha-KB stimulates peroxisome biogenesis and function

**Fig. 3:**
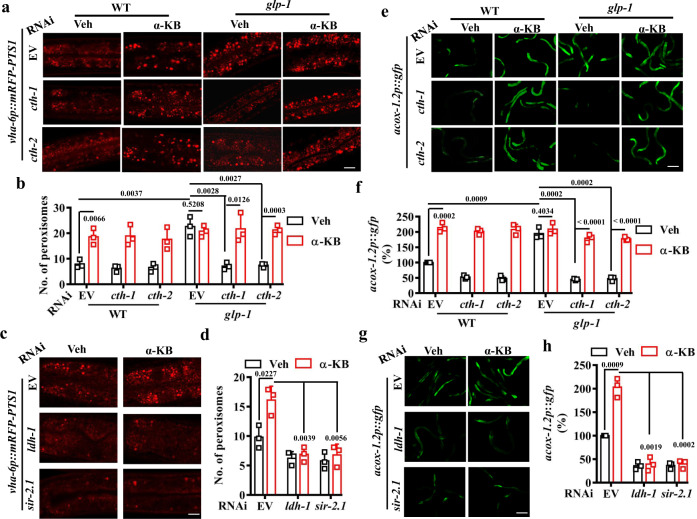
Alpha-KB promotes peroxisome function and biogenesis via the NAD^+^-SIR-2.1 pathway. **a** Representative images of peroxisomes in *glp-1*(*e2141ts*) mutant worms expressing *vha-6p::mRFP-PTS1*. Scale bars: 2.5 μm. Veh vehicle. **b** The number of peroxisomes was increased in *glp-1*(*e2141ts*) mutants. RNAi knockdown of either *cth-1* or *cth-2* inhibited this increase, which was rescued by supplementation with α-ketobutyrate (α-KB, 500 μM). Data were presented as mean values ± SEM of three independent experiments (*n* = 25 worms per experiment). **c** Representative images of peroxisomes in transgenic worms expressing *vha-6p::mRFP-PTS1* after treatment with α-KB. **d** Supplementation with α-KB (500 μM) increased the number of peroxisomes in worms, which was inhibited by RNAi knockdown of either *ldh-1* or *sir-2.1*. Data were presented as mean values ± SEM of three independent experiments (*n* = 25 worms per experiment). **e** Representative images of *acox-1.2p::gfp* in *glp-1*(*e2141ts*) worms. Scale bars: 150 μm. **f** Expression of *acox-1.2p::gfp* was upregulated in *glp-1*(*e2141ts*) mutants. RNAi knockdown of either *cth-1* or *cth-2* inhibited this increase, which was rescued by supplementation with α-KB (500 μM). Data were presented as mean values ± SEM of three independent experiments (*n* = 35 worms per experiment). **g** Representative images of *acox-1.2p::gfp* in worms after treatment with α-KB. **h** Supplementation with α-KB (500 μM) increased the expression of *acox-1.2p::gfp* in WT worms, which was inhibited by RNAi knockdown of either *ldh-1* or *sir-2.1*. Data were presented as mean values ± SEM of three independent experiments (*n* = 35 worms per experiment). *P* values throughout were calculated using a one-way ANOVA followed by a Student–Newman–Keuls test. Source data are provided as a Source Data file.

As mentioned above, we compared with the expression profiles of *glp-1*(*e2141ts*) mutants from two previous transcriptomic studies^20,28^ (Supplementary Fig. 1a). Among the overlapping genes from those two studies, both KEGG pathway and Gene Ontology enrichment analyses revealed that one of the top five overrepresented categories was “peroxisome” (Supplementary Fig. 1c). The “peroxisome” genes included peroxins (PEXs) required for peroxisomal protein import and proliferation (e.g., *prx-1*, *-2*, *-3*, *-5*, *-11*, -*12*, and -*19*)^45^, and peroxisomal genes required for fatty acid beta-oxidation (e.g., *acox-1.1*, -*1.2*, *-1.3*,- *1.4*, -*1.5*, -*1.6*, *-3*, *acs-2*, *-5*, -*13*, *-16*, -*17*, and *dhs-28*) (Supplementary Fig. 1d). These results suggest that germline removal probably enhances peroxisome biogenesis. To monitor whether peroxisome number and size were altered, we used a *C. elegans* peroxisome reporter in which mRFP was linked to a peroxisome-targeting sequence (PTS1) under control by an intestinal promoter (*vha-6p::mRFP-PTS1*)^31^. Confocal images were taken from the Int1 and Int2 cells (the first two anterior rings of the intestine) (Supplementary Fig. 6a). We found that germline-deficient animals displayed an increase in the abundance of mRFP puncta over WT animals (Fig. 3a, b). Meanwhile, the size of peroxisomes also increased in germline-deficient animals (Supplementary Fig. 6b). It should be noted that the punctate pattern was not observed in non-fluorescent strains (Supplementary Fig. 6a). RNAi knockdown of either *cth-1* or *cth-2* reduced the number and size of peroxisomes in *glp-1(e2141ts)* mutants back to those of WT worms (Fig. 3a, b and Supplementary Fig. 6b). Although supplementation with α-KB did not influence the number and size of peroxisomes in germline-deficient animals, it significantly restored the number and size of peroxisomes in these animals subjected to *cth-1* or *cth-2* RNAi (Fig. 3a, b and Supplementary Fig. 6b). Meanwhile, the number and size of peroxisomes were considerably increased in WT worms treated with α-KB (Fig. 3c, d and Supplementary Fig. 6c).

Although PTS1-labeled marker proteins, such as mRFP-PTS1 or GFP-SKL, have been used to determine the number and size of peroxisomes^31,46^, they actually represent peroxisomal import function^31^. We thus determined the number and size of peroxisomes using transgenic worms expressing *pmp-2p::pmp-2::mCherry*. PMP-2, the *C. elegans* homolog of mammalian PMP70, is a peroxisomal membrane protein^46^. We observed that both the number and size of peroxisomes were increased in both *glp-1(e2141ts)* mutants and α-KB-treated worms expressing *pmp-2p::pmp-2::mCherry* (Supplementary Fig. 7a–f). As PEX11 in yeast and mammals is involved in peroxisomal division and proliferation, we also determined the effect of α-KB on the number and size of peroxisomes by measuring the expression of *prx-11p::prx-11::gfp*, the *C. elegans* homolog of PEX11. We found that both the number and size of peroxisomes were increased in α-KB-treated worms expressing *prx-11p::prx-11::gfp* (Supplementary Fig. 8a–c). These results suggest that elevated α-KB levels via upregulation of *cth-1* and *cth-2* in *glp-1(e2141ts)* mutants or supplementation with α-KB in WT worms increase the number and size of peroxisomes.

Using qPCR, we determined the expression of the peroxisome-related genes, such as *prx-2*, *prx-19*, *acox-1.2*, *acox-3*, *acs-2*, and *acs-5*. Of these genes, the expressions of these peroxisome-related genes were significantly upregulated in *glp-1*(*e2141ts*) mutants, compared with those in WT worms (Supplementary Fig. 9a). Knockdown of *cth-1* or *cth-2* by RNAi reduced the expressions of these genes in *glp-1*(*e2141ts*) mutants back to those of WT worms. Utilizing transgenic worms expressing *acox-1.2p::gfp*, we found that the expression of *acox-1.2p::gfp* was substantially upregulated in *glp-1(e2141ts)* mutants (Fig. 3e, f). RNAi knockdown of *cth-1* or *cth-2* reduced the expression of *acox-1.2p::gfp* in *glp-1*(*e2141ts*) mutants back to those of WT worms. This increase in the expression of *acox-1.2p::gfp* was inhibited after the knockdown of *cth-1* and *cth-2* by RNAi. Although supplementation with α-KB did not influence the expression of *acox-1.2p::gfp* in *glp-1*(*e2141ts*) mutants, it markedly restored its expression in these animals subjected to *cth-1* or *cth*-2 RNAi (Fig. 3e, f). Finally, we found that α-KB treatment significantly upregulated the expression of *acox-1.2p::gfp* and *ctl-2*, encoding a peroxisomal catalase, in WT worms (Fig. 3g, h and Supplementary Fig. 9b). Taken together, these results suggest that peroxisome biogenesis and function are promoted by α-KB.

We asked the question of whether α-KB promoted peroxisome biogenesis by the production of NAD^+^. As α-KB can produce NAD^+^ via the lactate dehydrogenase^43,44^, we blocked the production of NAD^+^ by silencing *ldh-1*, which encoded the *C. elegans* lactate dehydrogenase. We found that knockdown of *ldh-1* by RNAi significantly inhibited the increase in the number and the size of peroxisomes (Fig. 3c, d and Supplementary Fig. 6c), and inhibited the expression of *acox-1.2p::gfp* in α-KB-treated WT worms (Fig. 3g, h). Next, knockdown of *sir-2.1* by RNAi suppressed the increase in either the number and size of peroxisomes (Fig. 3c, d and Supplementary Fig. 6c) or the expression of *acox-1.2p::gfp* in WT worms treated with α-KB (Fig. 3g, h). In addition, we tested the roles of NAD^+^ precursors, such as NAM, NMN, and nicotinamide riboside (NR)^12^. We found that supplementation with NAM, NMN, and NR considerably promoted the number and size of peroxisomes (Supplementary Figs. 10a–c, 11a–c), and upregulated the expression of *acox-1.2p::gfp* in WT worms (Supplementary Fig. 10d, e). Taken together, these results indicate that α-KB promotes peroxisome biogenesis and function by NAD^+^ and *sir-2.1*.

A previous study has demonstrated that AMP-activated protein kinase (AMPK) and caloric restriction promote longevity via remodeling mitochondrial and peroxisomal networks in worms^31^, raising the possibility that α-KB may function in mitochondria-peroxisome coordination. However, we found that α-KB did not delay the reduction in mitochondrial content with age (Supplementary Fig. 12a). Furthermore, RNAi knockdown of *aak-2*, which encodes catalytic subunits of AMPK, did not affect the lifespan of α-KB-treated WT animals (Supplementary Fig. 12b and Supplementary Data 1). More importantly, supplementation with α-KB did not affect the levels of phosphorylated AMPK(Thr172) in both worms and senescent IMR90 cells, a human lung fibroblast strain (Supplementary Fig. 12c, d). By contrast, the levels of phosphorylated AMPK(Thr172) were markedly higher in *glp-1(e2141ts)* mutants than those in WT worms (Supplementary Fig. 12c). Thus, lifespan extension by α-KB is not linked to maintenance of the mitochondrial networks mediated by AMPK.

### Peroxisome function and H_2_O_2_ production are required for α-KB-mediated lifespan extension

**Fig. 4:**
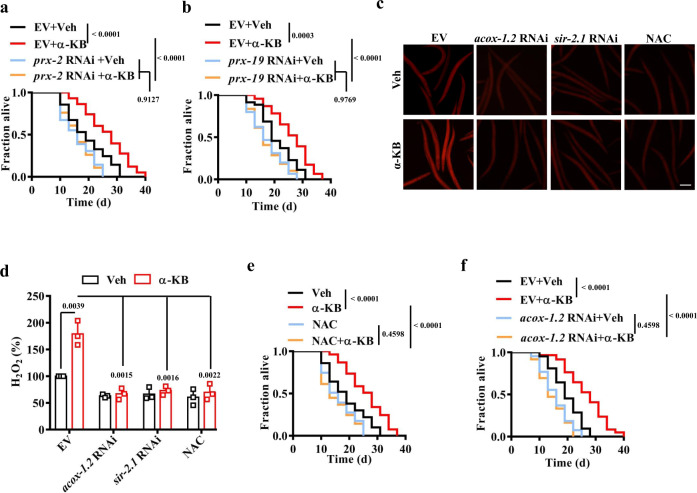
Peroxisome function and H_2_O_2_ production are involved in α-KB-mediated lifespan extension. **a**, **b** RNAi knockdown of *prx-2* (**a**) and *prx-19* (**b**) inhibited the lifespan extension of wild-type (WT) worms by treatment of α-ketobutyrate (α-KB, 500 μM). Veh vehicle. *P* values (**a**, **b**) were calculated using a log-rank test. **c** Representative images of H_2_O_2_ formation detected by fluorescence dye DCHP. Scale bars: 150 μm. **d** Quantification of fluorescence intensity. The levels of H_2_O_2_ were increased in WT worms treated with α-KB. RNAi knockdown of either *acox-1.2* or *sir-2.1* inhibited the levels of H_2_O_2_ in α-KB-treated WT worms. Data were presented as mean values ± SEM of three independent experiments (*n* = 35 worms per experiment). *P* values were calculated using a one-way ANOVA followed by a Student–Newman–Keuls test. **e** Supplementation with N-acetylcysteine (NAC) (5 mM) shortened the lifespan of WT worms treated with α-KB (500 μM). **f** Knockdown of *acox-1.2* by RNAi suppressed the lifespan of WT worms treated with α-KB (500 μM). *P* values (**e**, **f**) were calculated using a log-rank test. See survival statistics in Supplementary Data 1. Source data are provided as a Source Data file.

To determine whether the peroxisomal function is required for longevity, we silenced the peroxisomal genes, *prx-2* (the *C. elegans* homolog of mammalian peroxisome biogenesis factor 2; PEX2) and *prx-19* (the *C. elegans* homolog of mammalian peroxisome biogenesis factor 19; PEX19), which are involved in the peroxisomal import proteins^47^. In WT worms treated with α-KB or vehicle, RNAi knockdown of either *prx-2* or *prx-19* severely reduced the number of peroxisomes (Supplementary Fig. 13a, b). These results indicate that mRFP fails to import into peroxisomes. Moreover, the knockdown of *prx-2* and *prx-19* by RNAi reduced the lifespan of α-KB-treated WT worms back to that of WT worms treated with vehicle (Fig. 4a, b and Supplementary Data 1). Thus, peroxisome function is required for lifespan extension mediated by α-KB.

ROS are involved in life extension induced by germ-cell loss^6^. One of the products in the first step of peroxisomal fatty acid β-oxidation is H_2_O_2_, which is produced by acyl-CoA oxidases (ACOXs)^48^. We thus asked whether H_2_O_2_ production was increased when peroxisome biogenesis is enhanced. Using a fluorescence dye DCHP^49^, we found that the levels of H_2_O_2_ were increased in *glp-1*(*e2141ts*) mutants (Supplementary Fig. 14a, b). To determine which ACOX is involved in H_2_O_2_ formation, we screened the upregulated *acox* genes, including *acox-1.1*, *-1.2*, *-1.3*, *-1.4*, *-1.5*, *-1.6*, and *-3*, by RNAi. Of these seven genes, RNAi knockdown of *acox-1.2*, rather than the other six *acox* genes, significantly reduced the levels of H_2_O_2_ in *glp-1*(*e2141ts*) mutants (Supplementary Fig. 14c), suggesting that ACOX-1.2 is required for H_2_O_2_ production. Furthermore, supplementation with α-KB significantly promoted the production of H_2_O_2_ (Fig. 4c, d). We found that RNAi knockdown of *acox-1.2* not only inhibited H_2_O_2_ formation in WT worms, but also reduced the levels of H_2_O_2_ in WT α-KB-treated worms (Fig. 4c, d). Similar results were obtained by knockdown of *sir-2.1* by RNAi (Fig. 4c, d). These results suggest that H_2_O_2_ formation is induced by α-KB via ACOX-1.2.

To test whether the induction of H_2_O_2_ formation is involved in lifespan extension, an antioxidant N-acetylcysteine (NAC) was pre-treated with worms. H_2_O_2_ formation was markedly attenuated by NAC in α-KB-treated WT worms (Fig. 4c, d). Furthermore, supplementation with α-KB failed to extend the lifespan of either NAC- or *acox-1.2* RNAi-treated worms (Fig. 4e, f and Supplementary Data 1). Consistent with a previous observation^15^, NAC treatment shortened the lifespan of *glp-1*(*e2141ts*) mutants (Supplementary Fig. 15 and Supplementary Data 1). Although the knockdown of *acox-1.2* by RNAi also suppressed the lifespan of *glp-1*(*e2141ts*) mutants, it did not further reduce the lifespan of NAC-treated *glp-1(e2141ts)* mutants (Supplementary Fig. 15, Supplementary Data 1). Taken together, these results suggest that H_2_O_2_ production from peroxisomal fatty acid β-oxidation is crucial in α-KB-mediated lifespan extension.

### SKN-1 is involved in α-KB-induced lifespan extension

**Fig. 5:**
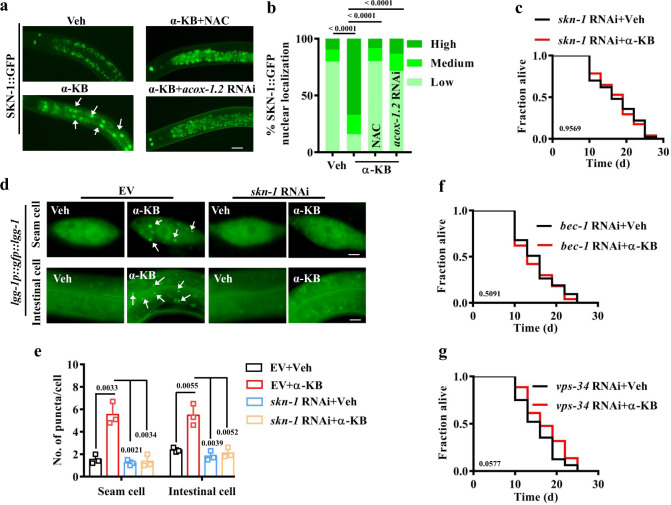
SKN-1-mediated autophagy is crucial for α-KB-induced lifespan extension. **a** Representative images of SKN-1::GFP translocation. Veh vehicle. Scale bars: 20 μm. **b** Quantification of SKN-1 nuclear accumulation. Supplementation with α-ketobutyrate (α-KB, 500 μM) promoted nuclear translocation of SKN-1::GFP in the intestine of worms, which was inhibited by treatment of *N*-acetylcysteine (NAC, 5 mM) or knockdown of *acox-1.2* by RNAi. Data were presented as mean values ± SEM of three independent experiments (*n* = 35 worms per experiment). *P* values were calculated using the Friedman test (with Dunn’s test for multiple comparisons). **c** Knockdown of *skn-1* by RNAi suppressed the lifespan of wild-type (WT) worms treated with α-KB (500 μM). *P* value was calculated using a log-rank test. **d** Representative images of autophagosomes (GFP::LGG-1 puncta) in the seam cells and intestinal cells of worms treated with α-KB (500 μM). Scale bars: 5 μm. **e** The numbers of GFP::LGG-1 puncta were counted. Data were presented as mean values ± SEM of three independent experiments (*n* = 35 worms per experiment). *P* values were calculated using a one-way ANOVA followed by a Student–Newman–Keuls test. **f**, **g** Knockdown of either *bec-1* (**f**) or *vps-34* (**g**) by RNAi significantly reduced the lifespan of WT worms treated with α-KB (500 μM). *P* values were calculated using a log-rank test. See survival statistics in Supplementary Data 1. Source data are provided as a Source Data file.

How might H_2_O_2_ from peroxisomal fatty acid β-oxidation extend lifespan? The oxidative stress-response transcription factors DAF-16 and SKN-1, which are involved in lifespan extension in germline-defective worms^20,50^, are potential candidates to serve as a downstream signal of H_2_O_2_. To test this hypothesis, we first determined whether α-KB activated SKN-1 and DAF-16. Utilizing transgenic worms expressing *skn-1p::skn-1::gfp* and *daf-16p::daf-16::gfp*, we observed that α-KB supplementation promoted the nuclear translocation of SKN-1, but not DAF-16 (Fig. 5a, b and Supplementary Fig. 16a, b). Furthermore, the expression of *gst-4p::gfp*, a SKN-1 target gene, was upregulated by α-KB supplementation (Supplementary Fig. 17a, b). Next, NAC treatment inhibited the nuclear translocation of SKN-1 in WT worms treated with α-KB (Fig. 5a, b). Furthermore, the knockdown of *acox-1.2* by RNAi also significantly suppressed the nuclear translocation of SKN-1 in worms treated with α-KB (Fig. 5b). These results suggest that H_2_O_2_ from peroxisomal fatty acid β-oxidation activates SKN-1.

SKN-1 is needed for longevity in *glp-1*(*e2141ts*) mutants^20^. Likewise, the knockdown of *skn-1* by RNAi also inhibited the lifespan extension in WT worms supplemented with α-KB (Fig. 5c and Supplementary Data 1). As induction of autophagy is required for longevity in *glp-1*(*e2141ts*) mutants^51,52^, we hypothesized that the activation of SKN-1 by α-KB might promote autophagy in worms. We thus determined autophagy levels by using transgenic worms carrying GFP::LGG-1. During autophagic processes, LGG-1/ATG8 is sequestered at the membrane of autophagosomes and condenses into puncta, which is a reliable indicator of autophagy^53,54^. The numbers of GFP::LGG-1-positive puncta in both the seam cells and the intestine in WT worms treated with α-KB were significantly higher than those in WT worms treated with vehicle (Fig. 5d, e). Knockdown of *skn-1* by RNAi substantially reduced the abundance of GFP::LGG-1 puncta in both the seam cells and the intestine in WT worms treated with α-KB (Fig. 5d, e). The accumulation of LGG-1 puncta may result from either induction of autophagy or a block in autophagy at a late step. To distinguish between these possibilities, we monitored both autophagosomes and autolysosomes using transgenic worms expressing *lgg-1p::mCherry::GFP::lgg-1*. In these worms, autophagosomes are positive for both GFP and mCherry, whereas autolysosomes are only positive for mCherry as the low pH quenched GFP in autolysosomes^51^ (Supplementary Fig. 18a, b). We observed mCherry single-positive autolysosome puncta and mCherry/GFP double-positive autophagosome puncta in seam cells and the intestine of control worms. Supplementation with α-KB significantly increased the numbers of both autophagosome and autolysosome puncta in worms subjected to empty vector, but not *skn-1* RNAi. These results indicate that α-KB promotes autophagic flux via SKN-1.

Next, we asked whether SKN-1 modulates autophagy transcriptionally. Using qPCR, we determined the expressions of autophagy-related genes, including those involved in autophagosome formation and autophagic flux, such as *atg-2*, *atg-11*, *lgg-2*, *vps-34*, *sqst-1/SQSTM1/p62*, and *unc-51/ULK1/ATG1*; lysosome-autophagosome fusion, such as *lmp-1/LAMP-1* and *lmp-2/LAMP-1* genes with lysosomal functions, such as subunits of vacuolar ATPases (*vha-10* and *vha-14*), cathepsins (*Y16B4A.2*, *asp-3/cathepsin D*, *asp-4 /cathepsin D*, and *tag-196/cathepsin F*), and *hlh-30/TFEB*. We found that most of these genes were upregulated in WT worms treated with α-KB (Supplementary Fig. 18c). Knockdown of *skn-1* by RNAi substantially reduced the transcription of most of these genes. These data suggest that the change in the expression of these autophagy- and lysosome-related genes is transcriptionally regulated by SKN-1.

To test whether autophagy is required for SKN-1-mediated lifespan extension, two autophagy-related genes *bec-1* and *vps-34* were silenced by RNAi. Both BEC-1 (the *C. elegans* homolog of ATG6/VPS30/beclin1) and VPS-34 (the *C. elegans* homolog of the yeast phosphatidylinositol 3-kinase Vps34p) are key regulators of autophagy initiation and progression^55,56^. We found that supplementation with α-KB failed to extend the lifespan of worms subjected to either *bec-1* or *vps-34* RNAi (Fig. 5f, g and Supplementary Data 1). These results indicate that SKN-1 is required for α-KB-induced lifespan extension by activating autophagy. As autophagy plays a crucial role in clearing damaged organelles, such as mitochondria, peroxisomes, and endoplasmic reticulum^57^, these results also raised the possibility that the induction of autophagy by SKN-1, which is activated by H_2_O_2_ derived from the peroxisome, could, in turn, degrades the peroxisome itself. To test this hypothesis, we determined the number of peroxisomes in worms treated with α-KB with increasing incubation time. However, we found that the number of peroxisomes was elevated at 24, 36, and 48 h after the addition of α-KB (Supplementary Fig. 19a, b). Furthermore, RNAi knockdown of *skn-1* did not affect the increase in the number of peroxisomes in worms after the addition of α-KB.

### Alpha-KB delays senescence in mammalian cells

**Fig. 6:**
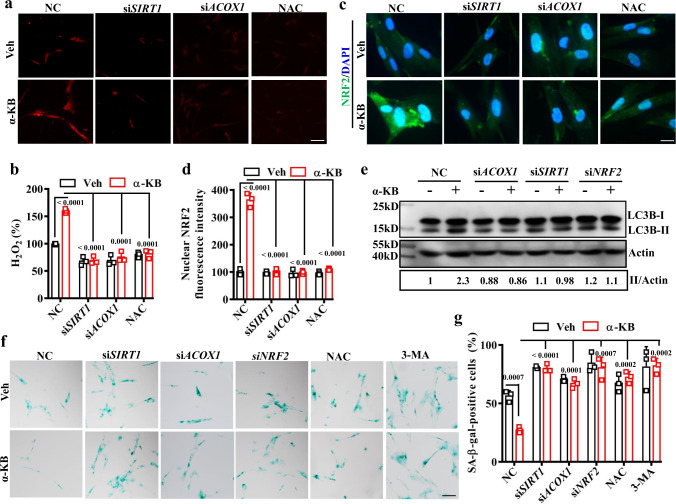
Alpha-KB delays senescence in mammalian cells. **a** Representative images of H_2_O_2_ formation detected by fluorescence dye DCHP in IMR90 cells. Scale bars: 15 μm. **b** Quantification of Fluorescence intensity. The H_2_O_2_ levels were increased in cells treated with α-ketobutyrate (α-KB, 500 μM) for 12 h, which was inhibited by RNAi knockdown of *SIRT1* or *ACOX1*, or pre-treatment with *N*-acetylcysteine (NAC, 5 mM). Data were presented as mean values ± SEM of three independent experiments. *P* values were calculated using a one-way ANOVA followed by a Student–Newman–Keuls test. **c** Representative images of immunofluorescence staining with anti-NRF2 antibodies. Scale bars: 5 μm. **d** Quantification of fluorescence intensity of NRF2 in the nucleus. Supplementation with α-KB (500 μM) induced nuclear accumulation of NRF2 in IMR90 cells, which was blocked by RNAi knockdown of *SIRT1* or *ACOX1*, or pre-treatment with NAC (5 mM). Data were presented as mean values ± SEM of three independent experiments. **e** Supplementation with α-KB (500 μM) increased the protein levels of LC3B-II in worms, which was blocked by RNAi knockdown of *SIRT1*, or *ACOX1*, or pre-treatment with NAC (5 mM). The blot shown here is typical of three independent experiments. Data were presented as mean values ± SEM of three independent experiments. *P* = 0.008, α-KB versus vehicle (veh); *P* = 0.0065, *ACOX1* RNAi + α-KB versus α-KB; *P* = 0.0057, *SIRT1* RNAi + α-KB versus α-KB; *P* = 0.0112, *NRF2* RNAi + α-KB versus α-KB. **f** Representative images of senescence-associated β-galactosidase (SA-β-gal) staining. Scale bars: 15 μm. **g** Quantification of SA-β-gal activity. Supplementation with α-KB (500 μM) reduced the SA-β-gal activity. Knockdown of *SIRT1*, *ACOX1*, or *NRF2* by RNAi, and pre-incubation of 3-MA (10 mM) abrogated the inhibitory effect of α-KB on SA-β-gal activity in cells treated with α-KB. Data were presented as mean values ± SEM of three independent experiments. *P* values throughout were calculated using a one-way ANOVA followed by a Student–Newman–Keuls test. Veh vehicle, NC negative control. Source data are provided as a Source Data file.

Since supplementation with α-KB extends lifespan in worms, we next asked whether such an intervention is beneficial for controlling cellular aging. For this, we tested the effect of α-KB on cell senescence using senescent IMR90 cells at population doubling (PD) 50. We found that treatment with α-KB led to a significant increase in H_2_O_2_ formation (Fig. 6a, b). However, knockdown of either *SIRT1* or *ACOX1* by RNAi or pre-treatment with NAC abrogated these effects of α-KB.

Next, we tested the effect of α-KB on nuclear location of NRF2, a feature that concurs with its activation^58^. Immunofluorescence analysis with anti-total NRF2 antibodies revealed that NRF2 exhibited a dual nuclear and cytoplasmic distribution in IMR90 cells under basal conditions (Fig. 6c, d). Treatment of α-KB promoted NRF2 translocation to the nucleus, which was inhibited by si-*SIRT1*, si-*ACOX1*, or NAC. We observed that phosphorylated NRF2 was exclusively located in the nucleus when detected using anti-phospho-NRF2(Ser40) antibodies (Supplementary Fig. 20a, b). Treatment of α-KB significantly increased the phosphorylated NRF2 levels in the nucleus, which was inhibited by si-*SIRT1*, si-*ACOX1*, or NAC. These results suggest that α-KB activates NRF2 mediated by the SIRT1-ACOX1 pathway.

Furthermore, we determined the effect of α-KB on autophagic activity by detecting LC3B-II levels. Upon the induction of autophagy, MAP1LC3B/LC3B (microtubule-associated protein 1 light chain 3b) is cleaved at the carboxyl terminus and conjugated to phosphatidylethanolamine. The conversion of cytosolic LC3B (LC3B-I) to phagophore-associated LC3B-II is a marker for autophagy. We observed a dramatic increase in the protein levels of LC3B-II in IMR90 cells treated with α-KB (Fig. 6e). However, knockdown of *SIRT1*, *ACOX1*, or *NRF2* by dsRNA significantly suppressed autophagy induced by α-KB. Finally, we determined the effect of α-KB on β-galactosidase activity, a well-established marker of cellular senescence^59^. The addition of α-KB reduced the β-galactosidase activity (Fig. 6f, g). Pre-treatment of NAC or RNAi knockdown of *SIRT1*, *ACOX1*, or *NRF2* significantly abrogated the inhibitory effect of α-KB on β-galactosidase (SA-β-gal) activity in IMR90 cells treated with α-KB. Likewise, pre-incubation of 3-methyladenine (3-MA)_ increased β-galactosidase activity and also abrogated the effect of α-KB (Fig. 6f, g). Together, these results suggest that α-KB also provides beneficial effects in mammalian cells by inducing autophagy through the SIRT1-ACOX1-H_2_O_2_- NRF2 pathway.

## Discussion

**Fig. 7:**
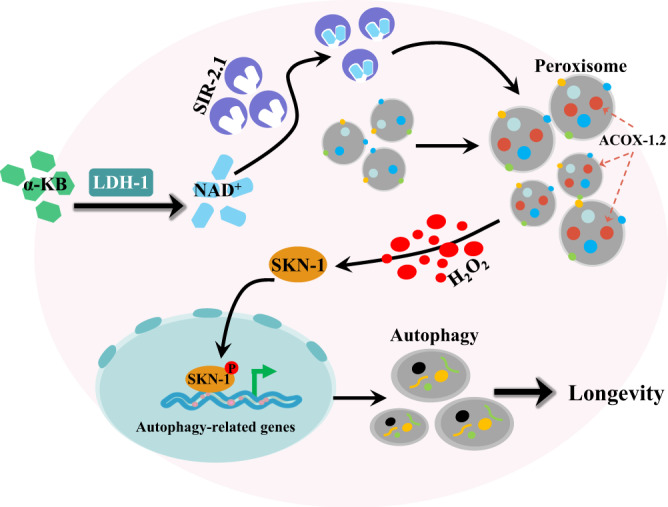
Schematic model for α-KB-mediated longevity. Supplementation with exogenous α-ketobutyrate (α-KB) increases the NAD^+^ contents via LDH-1. The NAD^+^-SIR-2.1/SIRT1 signaling enhances peroxisome function and biogenesis, thus leading to the upregulation of *acox-1.2*/*ACOX1*, a key gene in the peroxisomal fatty acid β-oxidation pathway. ACOX-1.2/ACOX1 promotes the production of H_2_O_2_, which in turn activates SKN-1/NRF2. This transcription factor induces autophagy by regulating the expressions of autophagic and lysosomal genes at a transcription level. Increased autophagic activity promotes longevity and delays cellular senescence.

Increased TSP activity promotes lifespan extension in both flies and worms^4,5^. As an intermediate in TSP, H_2_S functions to mediate longevity in yeast, worms, and fruit fly, and protect against hepatic ischemia-reperfusion injury^1,4,6,15,60,61^. The central finding in this study is that supplementation with exogenous α-KB, another intermediate in TSP, extends *C. elegans* lifespan and delays senescence in fibroblast cells. This longevity effect is likely mediated by the augmentation of NAD^+^ via LDH-1. This increase in substrate allows for SIR-2.1/SIRT1-dependent increases in peroxisome function and biogenesis, thus leading to upregulation of *acox-1.2/ACOX1*, a key gene in the peroxisomal fatty acid β-oxidation pathway. ACOX-1.2/ACOX1 enhances the production of H_2_O_2_, which subsequently activates SKN-1/NRF2. This transcription factor induces autophagy by driving the expression of autophagic and lysosomal genes, which in turn promotes longevity (Fig. 7). Our findings demonstrate a novel mechanism for extending lifespan that is mediated by an endogenous metabolite.

By mining two transcriptomic datasets^20,28^, we show that there are ~50 peroxisomal genes upregulated in the long-lived germline-deficient worms. In contrast, a proteome analysis shows that the abundance of ~30 peroxisomal proteins is reduced, and protein import into the peroxisome is compromised in old worms^30^. These omics data implicate an association between peroxisome and aging. However, until now, the exact role of peroxisome in aging and longevity remains largely unknown. While knockdown of peroxisomal genes *pex1*/*prx-1* and *pex13*/*prx-13* promotes lifespan in flies^32^, deletion of *pex5*/*prx-5* significantly shortens chronological lifespan in yeast^33^. In long-lived double null *drp-1*;*fzo-1* mutant worms, the size of peroxisomes is significantly increased^31^. Inhibition of peroxisomal function by silencing *pex5*/*prx-5* almost blocks lifespan extension by a double mutation in *drp-1;fzo-1*. In this study, supplementation with α-KB remarkably increases the abundance and size of peroxisomes, which is required for lifespan extension by α-KB. Thus, our findings support the idea that enhanced peroxisome function may represent a common signature associated with longevity.

During aging, the intracellular NAD^+^ levels are reduced in a variety of tissues in mammals^62–64^. The increase in intracellular NAD^+^ levels by supplementation of NAD^+^ precursors such as NAM and NMN, or activation of the de novo NAD^+^ synthesis pathway, improves lifespan and healthspan, and prevents age-associated metabolic dysfunction via a mechanism requiring SIRT1/SIR-2.1 in species ranging from mammals to worms^9–11,41^. Our data show that the long-lived *glp-1*(*e2141ts*) animals have higher NAD^+^ levels, which are attributed to increased endogenous α-KB by CTH-1 and CTH-2. Furthermore, supplementation with exogenous α-KB increases the production of NAD^+^, thus extending the lifespan in WT worms in a SIRT1/SIR-2.1-dependent manner. These results suggest that α-KB exhibits its beneficial effect on delaying senescence, at least in part, via the production of NAD^+^. The NAD^+^-SIRT1 pathway promotes longevity and prevents age-related physiological decline by inducing the mitochondrial unfolded protein response in worms and mammalian cells^41^. Moreover, NAD^+^ augmentation significantly extends lifespan and improves healthspan by promoting mitophagy via SIRT1/SIRT2.1 in worm and mouse models of Ataxia Telangiectasia^9^, a rare autosomal recessive disease, and in worm and fly models of Werner syndrome^65^, a human premature aging disease. In this study, our data demonstrate that supplementation with α-KB remarkably increases peroxisome function and biogenesis in WT worms in a SIRT2.1-dependent manner. Likewise, the NAD^+^ precursors, including NAM, NMN, and NR, also induces an increase in peroxisome function and biogenesis in worms. Thus, our study identifies enhanced peroxisome function as a downstream mechanism underlying NAD^+^-SIR-2.1-mediated longevity.

Although mitochondria are the main organelle responsible for ROS produced within cells, the peroxisome also generates ROS. In germline-deficient worms, the production of ROS is significantly elevated^15^. Besides these two sources of ROS (the mitochondrial ROS and DHE-reactive ROS produced by KRI-1)^15^, we show that H_2_O_2_ produced from the peroxisomal β-oxidation mediated by α-KB is another source of ROS in the germline-deficient worms. Accumulating evidence suggests that ROS may function as important modulators to promote lifespan in worms^15,23–26,66,67^. Interestingly, younger cells or worms are capable of generating more ROS than older individuals in response to environmental stresses^68^. Such redox-stress-response capacity is pivotal to maintain cellular redox and protein homeostasis. In the present study, α-KB-mediated H_2_O_2_ extends the lifespan in worms and delays cellular senescence by activating the ROS-sensitive transcription factor SKN-1/NRF2. SKN-1/NRF2 plays an important role in promoting longevity and delaying senescence in worms and mammals^58,69,70^; however, it is not known whether SKN-1 exhibits its effect on longevity by modulating autophagy. We show that SKN-1 activates autophagy by initiating a transcriptional program that coordinates the different steps of the autophagic pathway, thereby extending the lifespan in worms. As NRF2 has been proven to positively modulate autophagy in mammalian cells^71^, it is concluded that, like HLH-30/TFEB^52^, SKN-1/NRF2 is probably another master regulator of autophagy across species.

In conclusion, our findings present new insights into the mechanism underlying endogenous metabolite-mediated longevity. Besides α-KB, other endogenous metabolites, such as oxaloacetate^72^, ω-6 polyunsaturated fatty acids^73^, and α-ketoglutarate^74^, can also delay aging and extend lifespan, suggesting that the key to longevity is hidden in our body. The endogenous metabolites provide new approaches for the prevention and treatment of aging and aging-related diseases.

## Methods

### Nematode strains

Multiple mutants and transgenic strains used in this study included *glp-1(e2141ts)*, Strains BC14661 (sEx14661 [*rCes acox-1.2::GFP* + pCeh361]), CL2166 (dvIs19 [(pAF15)*gst-4p::GFP::NLS*]), *eat-2(ad1116), daf-2(e1370)*, *isp-1(qm150)*, and AM140 (rmls132[*unc-54p::Q35::YFP*]) were kindly provided by the Caenorhabditis Genetics Center (CGC; http://www.cbs.umn.edu/CGC), funded by NIH Office of Research Infrastructure Programs (P40 OD010440). The nematode strains VS10 (hjIs37 [*vha-6p::mRFP-PTS1* + *Cbr-unc-119(+)*]), DA2123 (adIs2122 [*lgg-1p::GFP::lgg-1* + *rol-6(su1006)*]), (hqIs181[*sdhl-1p::mtLs-gfp*]), MAH215 (sqIs11[*lgg-1p::mCherry::GFP:: lgg-1*]), *pmp-2p::pmp-2::mCherry*, and *prx-11p::prx-11::gfp* were kindly provided by Drs. Bin Liang (Yunnan University), Bin Qi (Yunnan University), Meng-Qiu Dong (National Institute of Biological Sciences, Beijing), Hong Zhang (Institute of Biophysics, Chinese Academy of Science), and Huanhu Zhu (ShanghaiTech University), respectively. Mutants were backcrossed three times into the N2 strain used in the laboratory. All strains were grown on nematode growth media (NGM) plates with *E. coli* OP50 at 20 °C^75^.

### Cell culture

The human lung fibroblast IMR90 cells were a gift from Dr. Xudong Zhao (Sichuan University West China Medical School) and obtained from the American Type Culture Collection (ATCC; CCL-186). The cells were grown in MEM with 1% sodium pyruvate, 1% penicillin-streptomycin, and 10% fetal bovine serum (Gibco, A3161001C) in a humidified, 5% CO_2_:95% air incubator at 37 °C. When attained 50 PD, IMR90 cells were treated with 500 μM of α-KB (MACKLIN, C10962521), 5 mM NAC (MACKLIN, N800425), or 10 mM 3-Methyladenine (MACKLIN, 5142-23-4). ddH2O or DMSO was used as a control.

### RNA interference for worms

RNAi bacterial strains containing targeting genes were obtained from the Ahringer RNAi library^76^. Briefly, *E. coli* strain HT115(DE3) expressing dsRNA corresponding to each worm gene was grown overnight in LB broth containing 100 μg/ml ampicillin at 37 °C, then spread onto NGM plates containing 100 μg/ml ampicillin and 5 mM isopropyl 1-thio-β-d-galactopyranoside (IPTG). The RNAi-expressing bacteria were then grown overnight at 25 °C. Synchronized L1 larvae were placed on the seeded RNAi plates at 20 °C until animals reached maturity. Young adult worms were used for further experiments.

### RNA interference for cells

All chemically synthesized siRNAs were obtained from Gene-Pharma Corporation. To silence the expression of *SIRT1*, *ACOX1*, or *NRF2* by RNAi, IMR90 cells were transfected at 50% confluence with 100 nM of siRNAs in Opti-MEM medium using Lipofectamine 2000 transfection agent (Thermo Fisher, 11668019). Gene silencing efficiency was confirmed by quantitative real-time PCR 72 h post-transfection. The following siRNAs were used (sequence of the sense strand): *SIRT1*, 5′-GGGAAAUGUAUUGGCAGUGUU-3′ (F), 5′-CACUGCCAAUACAUUUCCCUU-3′ (R); *ACOX1*, 5′-UCAUCUAAGAGACCUAGGCTT-3′ (F), 5′-GCCUAGGUCUCUUAGAUGATT-3′ (R); *NRF2*, 5′-UCAUCUAAGAGACCUAGGCTT-3′ (F), 5′-GCCUAGGUCUCUUAGAUGATT-3′ (R); negative control 5′-UUCUCCGAACGUGUCACGUTT-3′ (F), 5′-ACGUGACACGUUCGGAGAATT-3′ (R).

### DAVID enrichment analysis

We annotated two published transcriptomic datasets (GSE43864 and GSE63075)^20,28^ based on the Wormbase database, of version WS236 (www.wormbase.org). We searched for enrichment of KEGG (Kyoto Encyclopedia of Genes and Genomes) pathway gene sets and GO terms using the 3208 upregulated genes overlapped between these two transcriptomic datasets. Identify enriched GO terms and KEGG pathway of the overlapped genes of the expression profiles from these two datasets by Database for Annotation, Visualization, and Integrated Discovery (DAVID) version 6.7 (https://david.ncifcrf.gov/home.j sp)^77^. GO term or KEGG pathway with an adjusted *p* value <0.01 was defined as significantly changed.

### Drug treatment

NGM agar plates seeded with *E. coil* OP50 were supplemented with 200 and 500 μM of α-KB, or 200 and 500 μM of NAM (MERCK, 98-92-0), or 200 and 500 μM of NMN (MedChemExpress (MCE), HY-F0004), or 200 and 500 μM of NR (MedChemExpress(MCE), HY-123033A), or 5 mM NAC. ddH_2_O or DMSO was used as a control.

### Lifespan analysis

Synchronized populations of young adult worms were grown on NGM agar plates seeded with *E. coli* OP50 and tested drugs at 20 °C to score lifespan^37^. To test the effect of drugs on lifespan, the *E. coli* OP50-seeded NGM agar plates were supplemented with these drugs. The first day of adulthood of animals was recorded as day 1. Worms were transferred to new plates every day during their reproductive period. After reproduction ceases, worms were transferred every three days. The number of worms was counted every day. Worms that did not move when gently prodded and lacking pharyngeal pumping were marked as dead. Three plates of 50–60 worms per plate were tested per assay and all experiments were performed three times independently. Life spans for each assay are provided in Supplementary Data 1.

### Age-related phenotypic marker assays in worms

On the first day of adulthood, animals were placed on NGM agar plates containing *E. coli* OP50 until day 8 of adulthood and the following age-related phenotypes were scored. (1) Pharyngeal pumping was determined by counting the number of contractions in the terminal bulb of the pharynx in 30-s intervals. (2) Body bending was measured by counting the number of body bends in 30 s intervals. 20–30 worms were examined per assay in three independent experiments.

### Detection of H_2_S in worms

The endogenous H_2_S levels in germline-deficient animals were determined using a lysosome-targeted fluorescent chemodosimeter according to the previously described method^34^. Briefly, worms were incubated in Petri dishes filled with M9 buffer containing the fluorescent chemodosimeter (10 μM) at 20 °C for 3 h. The worms then were washed three times with M9 buffer and mounted in M9 onto microscope slides. The slides were viewed using a Zeiss Axioskop 2 Plus fluorescence microscope. The brighter the blue coloration, the stronger the fluorescence signal intensity. At least 30 worms were examined per assay in three independent experiments.

### Measurement of α-KB by LC/MS

The levels of α-KB in adult worms were determined by LC/MS. Briefly, after ~5 ml of packed worms were (Scanvac-Collsafe 110-4, LaboGene, Lynge, Denmark) for 48 h. The worm samples were then mixed with 2 ml of lysis buffer RIPA (Beyotime Institute of Biotechnology, Haimen, China), and gently ultrasonicated for 20 min. The mixture was centrifuged at 16,600×*g* for 30 min at 4 °C, and the supernatant was collected and then extracted with ethyl acetate. After the final centrifugation, the supernatant was collected, and analyzed by the Thermo Scientific Dionex Ultimate 3000 UHPLC system equipped with a Thermo high-resolution Q Exactive focus mass spectrometer (Thermo, Bremen, Germany). Samples were separated on a ZORBAX SB-C18 (4.6 mm × 250 mm, 5 μm particles; Agilent Technologies) with gradient elution. Ten microliters of samples were injected into the column using an autosampler. The mobile phase consisted of solvent A (water) containing 0.1% formic acid and solvent B (methanol), The flow rate of the mobile phase was 1 ml/min. The gradient program was as follows: 0–2 min (5% B), 2–8 min (5–15% B), 8–12 min (15–98% B), 12–16 min (98% B), 16–20 min (5% B). The instrument settings were as follows: capillary temperature of 320 °C, sheath gas flow rate of 35 (arbitrary units), an auxiliary gas flow rate of 15 (arb), spray voltage of −2.5 kV, full MS resolution of 70,000. Data were analyzed by using Thermo Scientific Xcaliber 4.4 software.

### Intestinal barrier function assay in worms

Intestinal barrier function was determined according to the method described previously^78^. Briefly, young adult worms were grown on standard NGM plates in the presence or absence of α-KB (500 μM) at 20 °C for 10 days. After being removed from the NGM plates, these worms were suspended in an M9 liquid medium containing *E*. *coli* OP50 (OD = ∼0.6), 5% food dye FD&C Blue No. 1 (Bis[4-(*N*-ethyl-*N*-3-sulfophenylmethyl) aminophenyl]-2-sulfophenylmethylium disodium salt) (AccuStandard, New Haven, CT), and further incubated for 6 h. After washing with M9 buffer four times, the worms were mounted in M9 onto microscope slides. The slides were viewed using a Zeiss Axioskop 2 Plus fluorescence microscope (Carl Zeiss, Jena, Germany) to measure the leakage of the dyes in the body cavity. The rate of body-cavity leakage was calculated as a percentage by dividing the number of worms with dye leakage by the number of total worms. Five independent experiments were carried out. In each experiment, at least 20 of worms were calculated.

### NAD^+^ measurements by LC/MS

NAD^+^ in worms was extracted by the method described previously^11^. Briefly, after ~1 ml of packed worms were freeze-dried in a freeze dryer (Scanvac-Collsafe 110-4, LaboGene, Lynge, Denmark) for 24 h. The worm samples then were extracted with 10% perchloric acid and neutralized in 3 M K_2_CO_3_ on ice. After centrifuged at 16,600 × *g* for 30 min at 4 °C to remove the insoluble residue, the supernatant was filtered, and analyzed by Thermo Scientific Dionex Ultimate 3000 UHPLC system equipped with a Thermo high-resolution Q Exactive focus mass spectrometer (Thermo, Bremen, Germany). Samples were separated on a ZORBAX SB-C18 (4.6 mm × 250 mm, 5 μm particles; Agilent Technologies) with gradient elution. Ten microliters of samples were injected into the column using an autosampler. The mobile phase consisted of solvent A (water) containing 0.1% formic acid and solvent B (methanol). The flow rate of the mobile phase was 1 ml/min. The method to measure NAD^**+**^ content is as follows: The gradient program was as follows: 0–2 min (2% B), 2–8 min (2–15% B), and 8–12 min (15–98% B), 12–16 min (98% B), 16–20 min (2% B). The instrument settings were as follows: capillary temperature of 350 °C, sheath gas flow rate of 35 (arbitrary units), an auxiliary gas flow rate of 15 (arb), spray voltage of 3.5 kV, full MS resolution of 70,000. The column temperature was set to 40 °C. Data were analyzed by using Thermo Scientific Xcaliber 4.4 software.

### Detection of H_2_O_2_ in worms

The H_2_O_2_ levels in worms were determined using a fluorescence dye, DCHP, according to the previously described method^49^. Briefly, worms were incubated in Petri dishes filled with M9 buffer containing DCHP (10 μM) at 20 °C for 5 h. Then, worms were washed with M9 buffer three times and mounted in M9 onto microscope slides. The slides were viewed using a Zeiss Axioskop 2 Plus fluorescence microscope. At least 30 worms were examined per assay in three independent experiments.

### Quantitative RT-PCR

Total RNA was extracted from worms with TRIzol Reagent (Invitrogen, Carlsbad, CA). Random-primed cDNAs were generated by reverse transcription of the total RNA samples with SuperScript II (Invitrogen). Quantitative real-time PCR analysis was performed using SYBR Premix (Takara, Dalian, China) on a Roche LightCycler 480 System (Roche Applied Science, Penzberg, Germany). The relative amount of each mRNA to *act-1* mRNA (an internal control) was calculated using the method described previously^79^. The primers used for qPCR assays are listed in Supplementary Data 2, respectively.

### Western blotting

Worms or cells were collected and lysed on ice for 30 min in lysis buffer RIPA (Beyotime Institute of Biotechnology, Haimen, China). The supernatant was obtained from cell lysates by centrifugation at 16,600 × *g* for 20 min at 4 °C and used for Western blot analysis. The proteins of lysates (50 μg per well) were separated on a 10% sodium dodecyl sulfate-polyacrylamide gel and then transferred to polyvinylidene fluoride membranes. The primary antibodies were anti-LC3B mAb (#3868, 1:3000 dilution, Cell Signaling Technology, Danvers, MA), anti-Phospho-AMPKα(Thr172) mAb (#2535, 1:1000 dilution, Cell Signaling Technology), anti-β-Actin mAb (#sc-8432, 1:4000 dilution, Santa Cruz Biotechnology, Santa Cruz, CA), and anti-AMPKα antibodies (#2532 S, 1:1000 dilution, Cell Signaling Technology). The secondary antibodies used were HRP-conjugated anti-mouse (#7074 s, 1:3000 dilution, Cell Signaling Technology) or anti-rabbit IgG (#7076p2, 1:3000 dilution, Cell Signaling Technology). An imaging system (Amersham Imager 600) was used for the documentation of the Western blot results.

### The quantification of peroxisome numbers and size

To determine the function and biogenesis of peroxisomes, we used the transgenic worms expressing *vha-6p::mRFP-PTS1*^31^, *pmp-2p::pmp-2::mCherry*, and *prx-11p::prx-11:gfp*^46^. Briefly, worms were collected and transferred in an Eppendorf tube filled with M9 buffer containing 4% formaldehyde at 20 °C for 5 min. After washing with M9 buffer three times, the worms were mounted in M9 onto microscope slides. The mRFP-PTS1, PMP-2::mCherry, and PRX-11::GFP fluorescence signals were acquired by Leica TCS SP8 STED confocal microscope (Leica Microsystems, Wetzlar, Germany) at 63x magnification. Confocal images were taken from the Int1 and Int2 cells (the first two anterior ring of the intestine). The diameters of peroxisomes were 0.1–1.2 μm^80^, as a standard for quantifying the number and size of peroxisomes. At least 20 worms were examined per assay in three independent experiments.

### Scoring of SKN-1 and DAF-16 nuclear accumulation

Young adult worms expressing *skn-1p::skn-1::gfp* or *daf-16p::daf-16::gfp* were grown on NGM agar plates containing *E. coil* OP50. After being treated with 500 μM α-KB for 24 h, the worms were immediately mounted in M9 onto microscope slides. The slides were viewed using a Zeiss Axioskop 2 Plus fluorescence microscope. The status of DAF-16::GFP localization was categorized as cytosolic localization or nuclear localization when localization was observed throughout the body from head to tail^81^. When nuclear localization was visible, but not completely throughout the body, the status of DAF-16::GFP localization was characterized as intermediate localization. At least 30 worms were examined per assay in three independent experiments. SKN-1::GFP accumulation in intestinal nuclei was scored as previously described^82^. “High” indicates that a strong SKN-1::GFP signal was present in all nuclei, “medium” indicates that nuclear SKN-1::GFP was detected in both anterior and poster intestinal nuclei, and “low” indicates that nuclear SKN-1::GFP was barely detectable in either anterior or posterior intestinal nuclei.

### Autophagy analysis

Young adult worms expressing GFP::LGG-1 were grown on NGM agar plates containing *E. coil* OP50 in the presence or absence of 500 μM α-KB. After 24 h of growth, the worms were immediately mounted in M9 onto microscope slides. The slides were viewed using a Zeiss Axioskop 2 Plus fluorescence microscope. GFP::LGG-1 positive puncta were counted in the seam cells or the intestine. At least 30 worms were examined per assay in three independent experiments.

### Microscopy

The transgenic worms carrying *acox-1.2p::gfp* or *gst-4p::gfp* were treated with 500 μM α-KB for 12 and 24 h. The worms were mounted in M9 onto microscope slides for detecting fluorescent signals. The slides were viewed using a Zeiss Axioskop 2 Plus fluorescence microscope. At least 30 worms were examined per assay in three independent experiments. The fluorescence intensity was analyzed using ImageJ2×V2.1.4.8 (NIH).

### Immunofluorescence

Cells were fixed with 4% paraformaldehyde (PFA) for 10 min at room temperature. After washed with PBS three times and were treated with PBS containing 0.1% Triton X-100 for 15 min, the cells were permeabilized, and blocked with PBST containing 5% fetal bovine serum for 90 min at room temperature. Then the cells were immunostained with anti-NRF2 mAb (#2F6C6, 1:500 dilution, Cusabio Technology Llc, Huston, TX) or anti-Phospho-NRF2(Ser40) antibodies (PA5-67520, 1:500 dilution, Thermo Fisher Scientific) overnight at 4 °C. After washed three times with 0.1% Tween-20 in PBS (PBST), these cells were incubated with Alexa Fluor 405 anti-mouse IgG (H + L) (A-55057, 1:200 dilution, Thermo Fisher Scientific) or Alexa Fluor 488 anti-Rabbit IgG (H + L) (A-21206, 1:200 dilution, Thermo Fisher Scientific) for 1 h. Then, the slides were washed three times with PBST and stained with 1 μg/ml of 4,6-diamidino-2-phenylindole (DAPI) for 30 min to detect nuclei. Images were acquired using a Zeiss Axioskop 2 plus fluorescence microscope (Carl Zeiss, Jena, Germany).

### Senescence-associated β-galactosidase staining

Senescence-associated SA-β-gal staining was performed according to the method described previously^83^. Briefly, IMR90 cells were fixed in 2% formaldehyde and 0.2% glutaraldehyde at room temperature for 10 min, and stained in freshly prepared staining solution (all reagents were from Cell Senescence β-galactosidase Staining Kit, Beyotime, Haimen) at 37 °C overnight. Images were taken and the percentages of SA-β-gal-positive cells were counted and determined using Image J. Each group had three biological replicates.

### Statistical analysis

All statistical tests were two-sided, and values of *P* < 0.05 were considered statistically significant. Differences in survival rates were analyzed using the log-rank test. Differences in gene expression, mRNA and protein levels, the contents of metabolites and H2O2, the percentages of SA-β-gal-positive cells, the numbers of GFP::LGG-1 positive puncta, and fluorescence intensity were assessed by performing one-way ANOVA followed by the Student–Newman–Keuls test or the Student’s *t*-test. Differences in the distribution of SKN-1 were analyzed using the Friedman test (with Dunn’s test for multiple comparisons) or the Wilcoxon signed-rank test. To test for significant overlap between different gene lists, Fisher’s exact test was used. Data were analyzed using GraphPad Prism 7 (GraphPad Software Inc., La Jolla, CA).

### Reporting summary

Further information on research design is available in the Nature Portfolio Reporting Summary linked to this article.

## Supplementary information

Supplementary Information

Description of Additional Supplementary Files

Supplementary Data 1

Supplementary Data 2

Reporting Summary

## Data Availability

The data supporting the findings of this study are available within the article and its supplementary materials. Lifespan data were collected in Supplementary Data 1. The primers used for qRT-PCR are listed in Supplementary Data 2. The transcriptome data that support the findings of this study are available in a publicly accessible repository from the GEO under accession GSE43864 and GSE63075. Source data are provided with this paper.

## Source data

Source Data
