# Supplementary material for: The metabolite alpha-ketobutyrate extends lifespan by promoting peroxisomal function in C. elegans
Source: Nat Commun. 2023 Jan 16;14:240. doi: 10.1038/s41467-023-35899-1 (PMC9842765; doi:10.1038/s41467-023-35899-1)
Supplement: Supplementary file 1 — Supplementary Information [file 41467_2023_35899_MOESM1_ESM.pdf]

Supplementary Information

The metabolite alpha-ketobutyrate extends lifespan by promoting peroxisomal function

Nan Wu, Yi-Cheng Ma, Xin-Qian Gong, Pei-Ji Zhao, Yong-Jian Jia, Qiu Zhao, Jia-Hong Duan, Cheng-Gang Zou

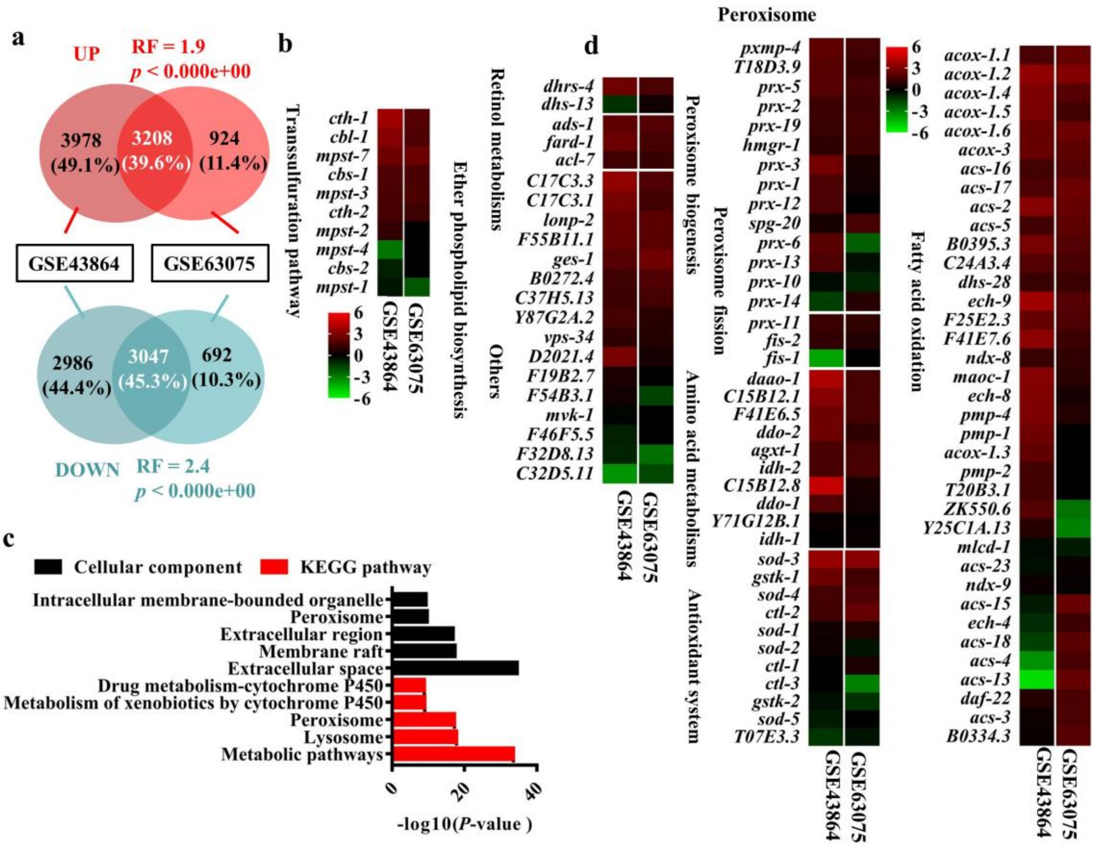

Supplementary Figure 1. The peroxisome category is overrepresented in two existing transcriptome data of *glp-1*(*e2141ts*) mutants (GSE43864 and GSE63075).

a There were 3208 up-regulated genes and 3047 down-regulated genes overlapped between these two datasets. *P*-values were calculated using a Fisher's exact test. The representation factor (RF) is the number of overlapping genes divided by the expected number of overlaping genes drawn from two independent groups. A representation factor > 1 indicates more overlap than expected of two independent groups. **b** The expression pattern of major genes in the transsulfuration pathway in these two datasets (GSE43864 and GSE63075). Color coding reflects relative representation in RNA-seq data (Log_2(FC)), with red and green indicating increased and decreased expression, respectively. **c** Both KEGG pathway and Gene Ontology enrichment analyses revealed that the peroxisome category was overrepresented in *glp-1(e2141ts)* mutants. The enrichment *P* value of each term was transformed to a -log10 (*P*-value). Only pathways with adjusted *P* value < $1E^{-9}$ are shown. For multiple comparisons, adjustments were made with Benjamini–Hochberg (BH) method. **d** These up-regulated

peroxisome-related genes were involved in peroxisome biogenesis, peroxisome fission, retinol metabolism, ether phospholipid biogenesis, fatty acid β-oxidation, antioxidant system, and amino acid metabolism. Color coding reflects relative representation in RNA-seq data (Log_2(FC)), with red and green indicating increased and decreased expression, respectively.

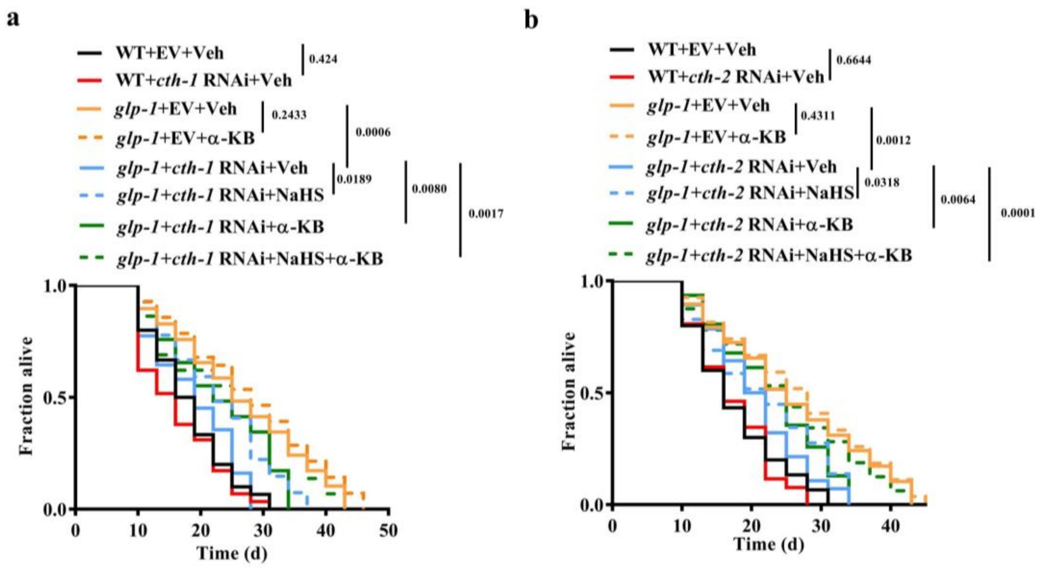

Supplementary Figure 2. *cth-1* and *cth-2* contribute to lifespan extension in *glp-1(e2141ts)* mutants via both α-KB and H_2S.

a, **b** RNAi knockdown of *cth-1* (**a**) or *cth-2* (**b**) shortened the lifespan of *glp-1(e2141ts)* mutants, which was partially rescued by supplementation with either α-KB (500 μM) or NaHS (300 μM). However, supplementation with both α-KB and NaHS fully restored the lifespan of *glp-1(e2141ts)* mutants subjected to either *cth-1* or *cth-2* RNAi. *P*-values were calculated using log-rank test. See survival statistics in Supplementary Data 1. Source data are provided as a Source Data file.

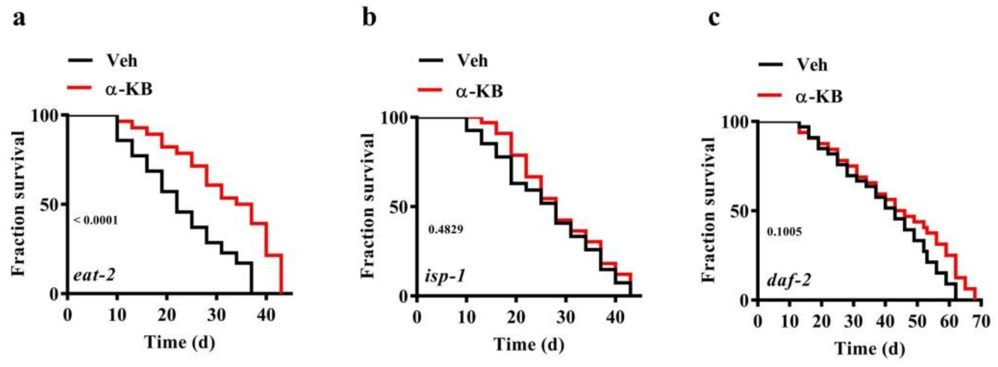

Supplementary Figure 3. Effect of α-KB on the lifespan of multiple longevity mutants.

a-c Supplementation with α-KB (500 μM) extended the lifespan of *eat-2(ad1116)* (**a**)**,** but not *isp-1(qm150)* (**b**) and *daf-2(e1370)* **(c)** mutants. *P*-values were calculated using log-rank test. See survival statistics in Supplementary Data 1. Source data are provided as a Source Data file.

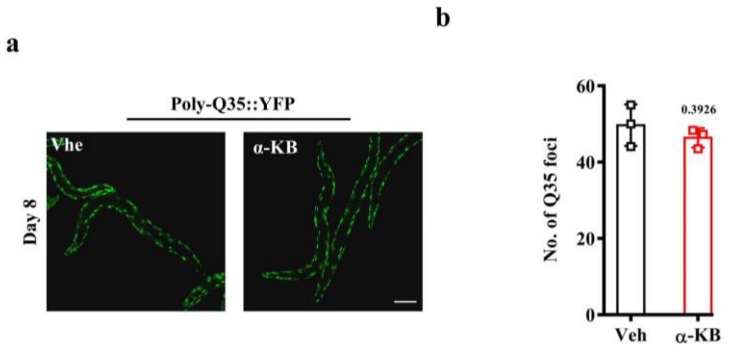

Supplementary Figure 4. Supplementation with α-KB does not affect the number of Poly-Q35::YFP aggregates in body wall muscle cells.

a Representative images of Poly-Q35::YFP aggregates in worms treated with α-KB (500 μM) on Day 8. A similar pattern of expression was observed in three independent experiments. Scale bars: 100 μm. **b** Quantification of GFP levels. Data are presented as mean values ± SEM of three independent experiments (n = 25 per experiment). *P*-value was calculated using the two sample t-test. Source data are provided as a Source Data file.

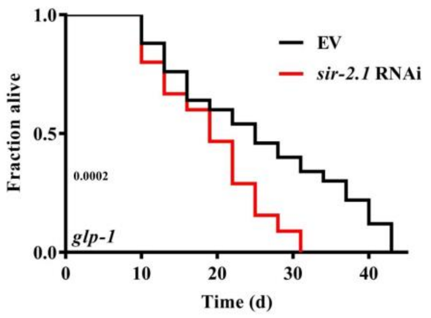

Supplementary Figure 5. RNAi knockdown of *sir-2.1* shortens the lifespan of *glp-1(e2141ts)* mutants.

P-value was calculated using log-rank test. See survival statistics in Supplementary Data 1. Source data are provided as a Source Data file.

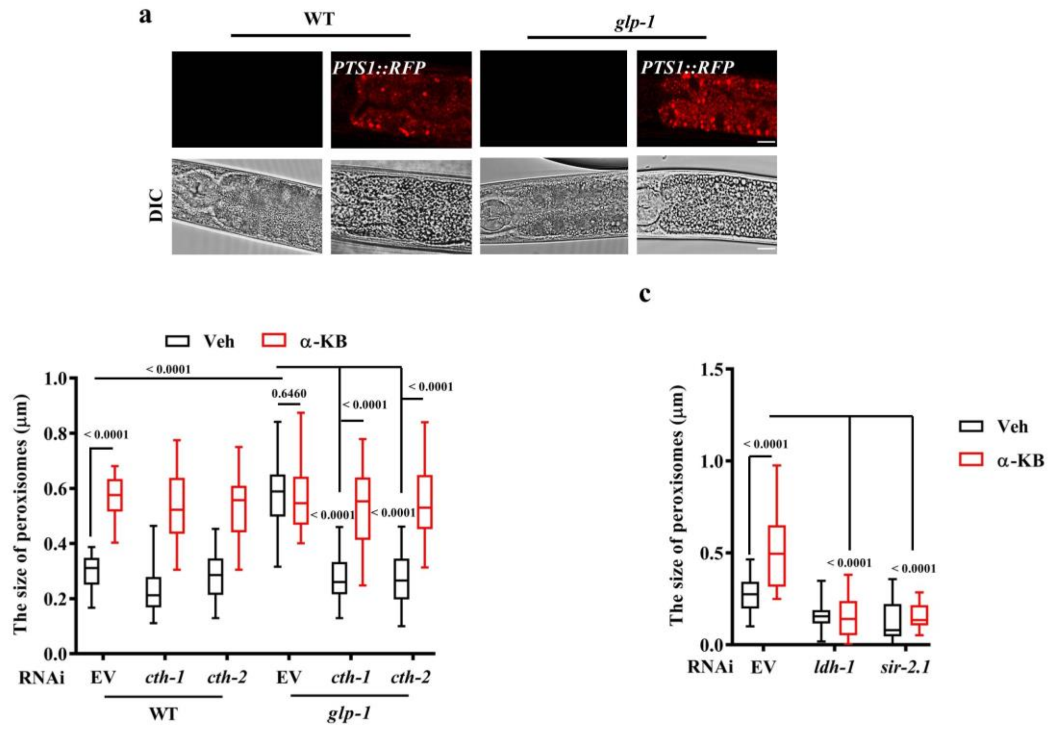

Supplementary Figure 6. Alpha-KB supplementation increases the number and size of peroxisomes in worms via the LDH-1-SIR-2.1 pathway.

a Confocal images were taken from the Int1 and Int2 cells (the first two anterior ring of the intestine) in transgenic worms expressing *vha-6p::mRFP-PTS1*. The punctate pattern was observed in the transgenic worms, rather than non-fluorescent strains. A similar pattern of expression was observed in three independent experiments. Scale bars: 2.5 μm. **b** The size of peroxisomes was increased in *glp-1*(*e2141ts*) mutants. RNAi knockdown of either *cth-1* or *cth-2* inhibited this increase, which was25 rescued by supplementation with α-KB (500 μM). Data are presented as mean values +/- SEM of three independent experiments (n = 10 worms per experiment). **c** Supplementation with α-KB increased the size of peroxisomes in wild type (WT) worms, which was inhibited by RNAi knockdown of either *ldh-1* or *sir-2.1*. Data are presented as mean values ± SEM of three independent experiments (n = 10 worms per experiment). The Whiskers connect the minimum and the maximum values to the Box, the line in the box marks the median, and upper and lower box border marks interquartile range (**b, c**), *P*-values throughout were calculated using a one-way ANOVA followed by a Student-Newman-Keuls test. Source data are provided as a Source Data file.

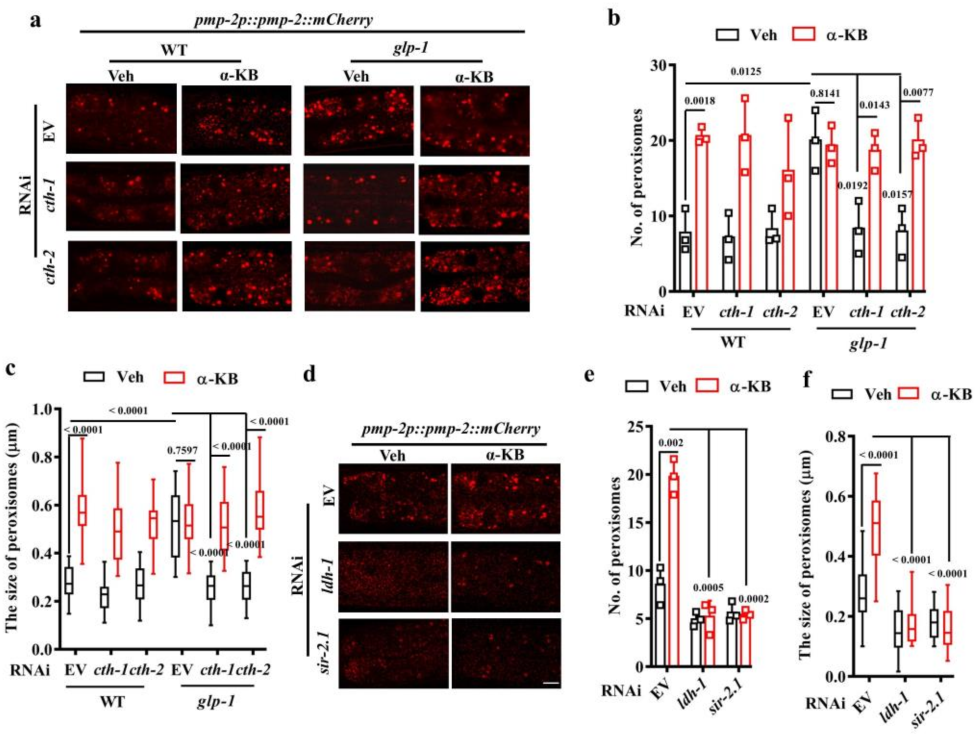

Supplementary Figure 7. Alpha-KB supplementation increased the number and size of peroxisomes in worms expressing *pmp-2p::pmp-2::mCherry*.

a, d Representative images of peroxisomes in worms expressing *pmp-2p::pmp-2::mCherry*. A similar pattern of expression was observed in three independent experiments. Scale bars: 2.5 μm. **b** The number of peroxisomes was increased in *glp-1*(*e2141ts*) mutants. RNAi knockdown of either *cth-1* or *cth-2* inhibited this increase, which was rescued by α-KB (500 μM). Data are presented as mean values ± SEM of three independent experiments (n = 25 worms per experiment). **c** The size of peroxisomes was increased in *glp-1*(*e2141ts*) mutants. Data are presented as mean values ± SEM of three independent experiments (n = 10 worms per experiment). **e** Supplementation with α-KB increased the number of peroxisomes in WT worms, which was inhibited by RNAi knockdown of either *ldh-1* or *sir-2.1*. Data are presented as mean values ± SEM of three independent experiments (n = 25 worms per experiment). **f** Supplementation with α-KB increased the size of peroxisomes in worms. Data are presented as mean values ± SEM of three independent experiments (n = 10 worms per experiment). The Whiskers connect the minimum and the maximum values to the Box, the line in the box marks the median, and upper and lower box border marks interquartile range (**c, f**). *P*-values throughout were calculated using a one-way ANOVA followed by a Student-Newman-Keuls test. Source data are provided as a Source Data file.

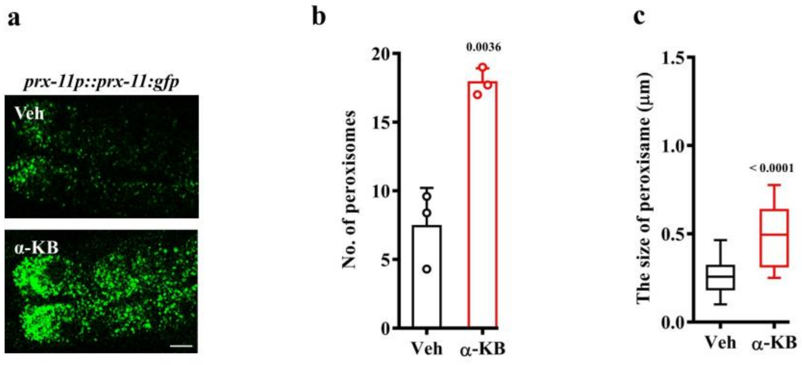

Supplementary Figure 8. Alpha-KB supplementation increased the number and size of peroxisomes in worms expressing *prx-11p::prx-11::gfp*.

a Confocal microscopy analysis of peroxisomes in worms expressing *prx-11p::prx-11::gfp*.. A similar pattern of expression was observed in three independent experiments. Scale bars: 5 μm. **b, c** Quantification of fluorescent intensity in **a**. **b** Supplementation with α-KB (500 μM) increased the number of peroxisomes in WT worms. Data are presented as mean values ± SEM of three independent experiments (n = 25 worms per experiment). **c** Supplementation with α-KB increased the size of peroxisomes in WT worms. Data are presented as mean values ± SEM of three independent experiments (n = 10 worms per experiment). The Whiskers connect the minimum and the maximum values to the Box, the line in the box marks the median, and upper and lower box border marks interquartile range. *P*-values throughout were calculated using the two sample t-test. Source data are provided as a Source Data file.

a

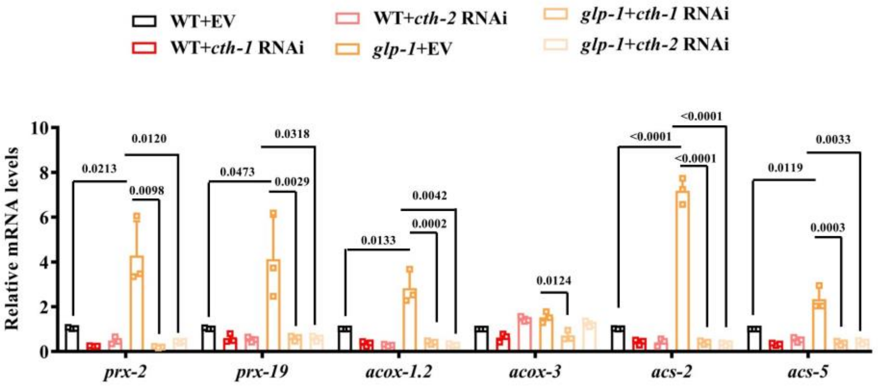

b

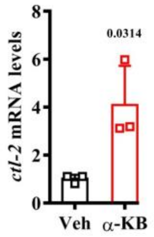

Supplementary Figure 9. The expressions of these peroxisome-related genes in worms.

a The mRNA levels of these peroxisome-related genes were up-regulated in *glp-1*(*e2141ts*) mutants, which were dependent on CTH-1 and CTH-2. **b** The mRNA levels of *ctl-2* were significantly up-regulated in worms treated by α-KB. Data are presented as mean values ±SEM of three independent experiments. *P*-values throughout were calculated using a one-way ANOVA followed by a Student-Newman-Keuls test or the two sample t-test. Source data are provided as a Source Data file.

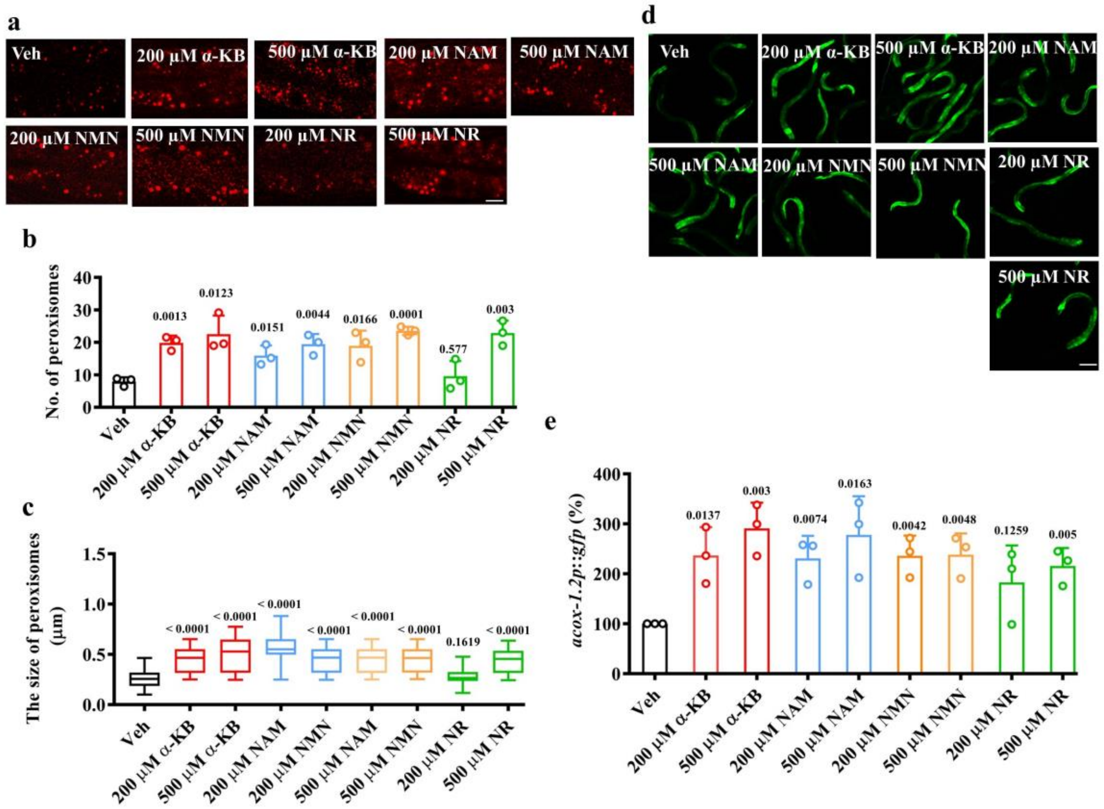

Supplementary Figure 10. NAD$^+$ precursors promote peroxisome function and biogenesis in WT worms.

a Representative images of peroxisomes in worms expressing *vha-6p::mRFP-PTS1*. A similar pattern of expression was observed in three independent experiments. Scale bars: 2.5 μm. **b** Supplementation with α-KB and NAD$^+$ precursors, such as NAM, NMN, and NR, increased the number of peroxisomes in WT worms. Data are presented as mean values ± SEM of three independent experiments (n = 25 worms per experiment). **c** Supplementation with α-KB, NAM, NMN, and NR increased the size of peroxisomes in WT worms. Data are presented as mean values ± SEM of three independent experiments (n = 10 worms per experiment). The Whiskers connect the minimum and the maximum values to the Box, the line in the box marks the median, and upper and lower box border marks interquartile range. **d** Representative images of *acox-1.2p::gfp*. Scale bars: 150 μm. **e** Expression of *acox-1.2p::gfp* was up-regulated in worms treated by α-KB, NAM, NMN, and NR. Data are presented as mean values ± SEM of three independent experiments (n = 35 worms per experiment). *P*-values throughout were calculated using a one-way ANOVA followed by a Student-Newman-Keuls test. Source data are provided as a Source Data file.

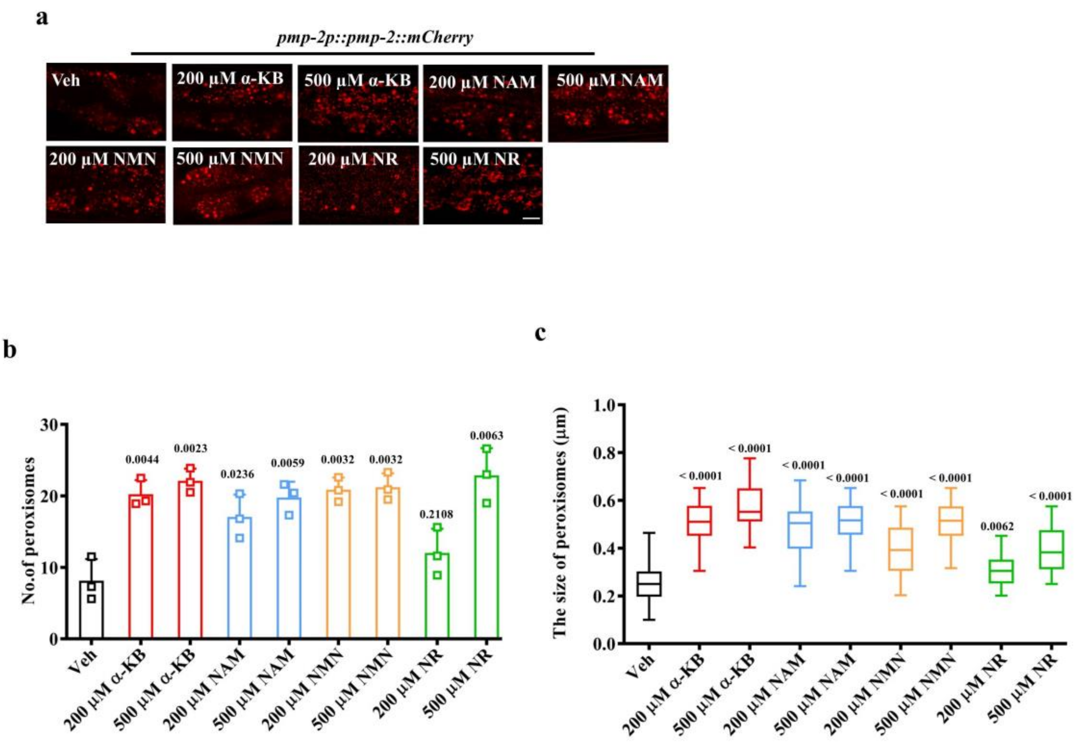

Supplementary Figure 11. NAD$^+$ precursors promote peroxisome function and biogenesis in worms expressing *pmp-2p::pmp-2::mCherry*.

a Representative images of peroxisomes in worms expressing *pmp-2p::pmp-2::mCherry*. A similar pattern of expression was observed in three independent experiments. Scale bars: 2.5 µm. **b** Supplementation with α-KB and NAD$^+$ precursors, such as NAM, NMN, and NR, increased the number of peroxisomes in WT worms. Data are presented as mean values ± SEM of three independent experiments (n = 25 worms per experiment). **c** Supplementation with α-KB, NAM, NMN, and NR increased the size of peroxisomes in WT worms. Data are presented as mean values ± SEM of three independent experiments (n = 10 worms per experiment). The Whiskers connect the minimum and the maximum values to the Box, the line in the box marks the median, and upper and lower box border marks interquartile range. *P*-values throughout were calculated using a one-way ANOVA followed by a Student-Newman-Keuls test. Source data are provided as a Source Data file.

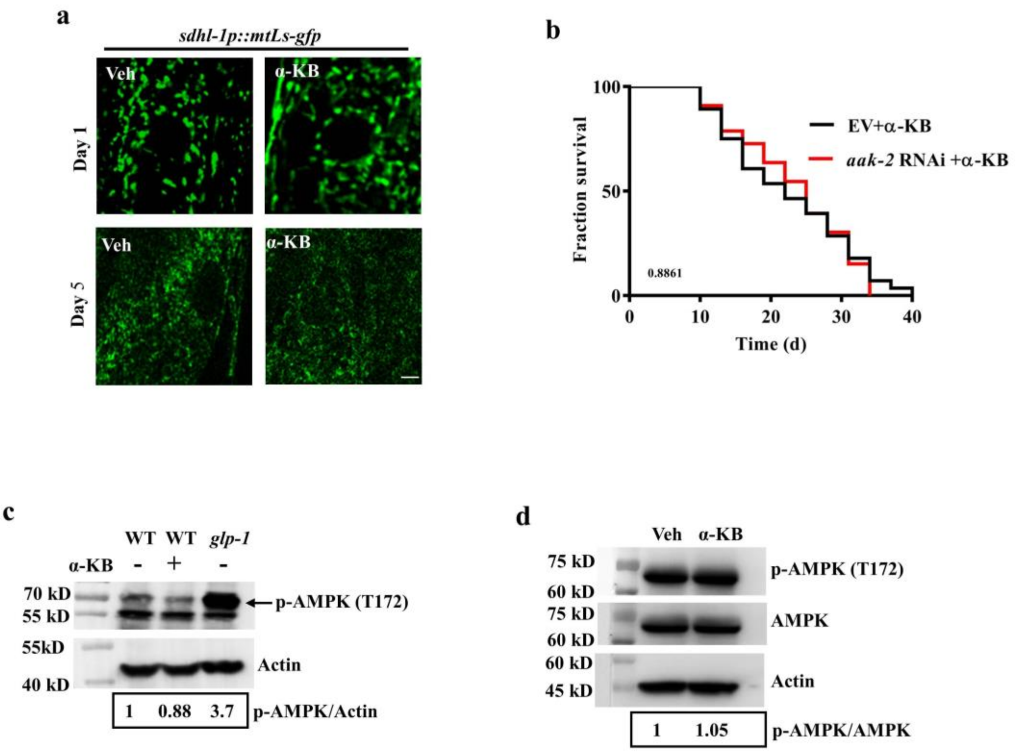

Supplementary Figure 12. Lifespan extension by α-KB is not due to maintenance of the mitochondrial networks mediated by AMPK.

a Representative images of mitochondria in worms expressing sdhl-1p::mtLs-gfp. Scale bars: 5 μm. Supplementation with α-KB did not delay the reduction in mitochondrial content with age. A similar pattern of expression was observed in three independent experiments. Scale bars: 5 μm. **b** RNAi knockdown of *aak-2* did not affect the lifespan of α-KB-treated WT animals. *P*-value was calculated using log-rank test. See survival statistics in Supplementary Data 1. **c, d** Supplementation with α-KB (500 μM) did not affect the levels of phosphorylated AMPK (Thr172) in worms (**c**) and IMR90 cells (**d**). The blot shown here is typical of three independent experiments. Data are presented as mean values ± SEM of three independent experiments. *P*=0.7054, WT+α-KB versus WT; *P*= 0.0183, *glp-1* versus WT (**c**). *P*= 0.7893, α-KB versus vehicle (**d**). *P*-values were calculated using the two sample t-test. Source data are provided as a Source Data file.

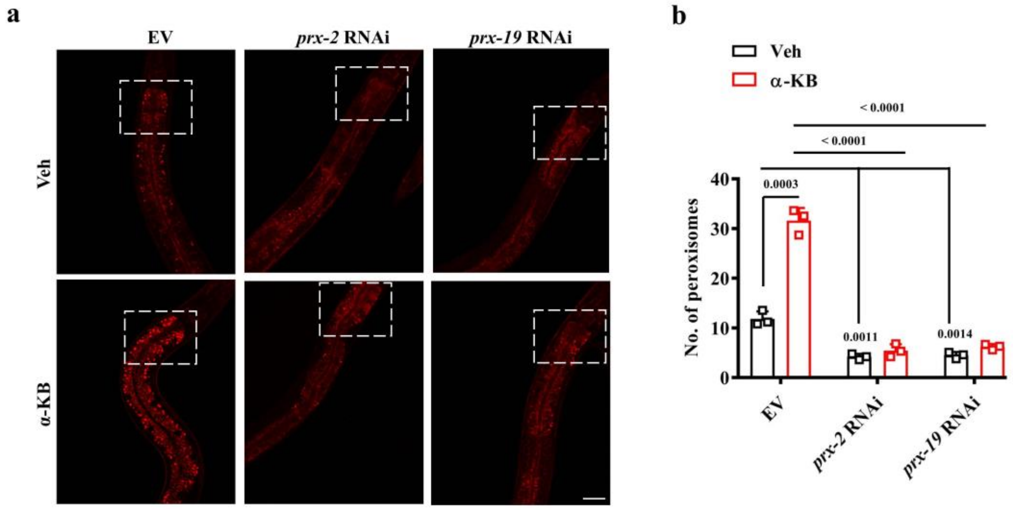

Supplementary Figure 13. RNAi knockdown of either *prx-2* or *prx-19* blocks peroxisomal protein import.

a Representative images of peroxisomes in worms expressing *vha-6p::mRFP-PTS1*. A similar pattern of expression was observed in three independent experiments. Scale bars: 25 μm. **b** RNAi knockdown of either *prx-2* or *prx-19* severely reduced the number of peroxisomes in WT worms treated with α-KB or vehicle. Data are presented as mean values ± SEM of three independent experiments (n = 25 worms per experiment). *P*-values throughout were calculated using a one-way ANOVA followed by a Student-Newman-Keuls test. Source data are provided as a Source Data file.

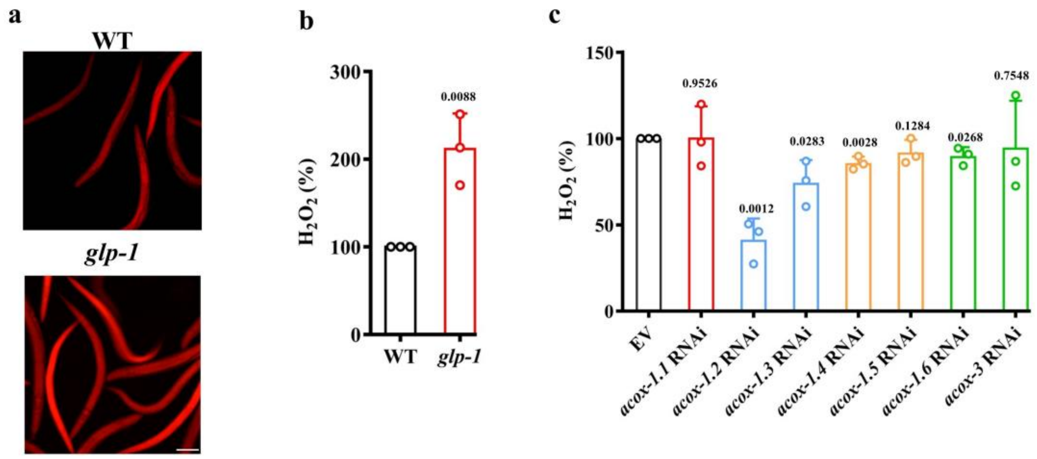

Supplementary Figure 14. H_2O_2 formation is mediated by *acox-1.2*.

a Representative images of H_2O_2 formation detected by fluorescence dye DCHP in *glp-1(e2141ts)* mutants. A similar pattern of fluorescence was observed in three independent experiments. Scale bars: 100 μm. **b** Quantification of Fluorescence intensity in **a**. The levels of H_2O_2 were increased in *glp-1(e2141ts)* mutants. Data are presented as mean values ± SEM of three independent experiments (n = 35 worms per experiment). *P*-values were calculated using the two sample t-test. **c** RNAi knockdown of *acox-1.2*, but not other acox genes, significantly reduced H_2O_2 formation in *glp-1(e2141ts)* mutants. Data are presented as mean values ± SEM of three independent experiments (n = 35 worms per experiment). *P*-values throughout were calculated using a one-way ANOVA followed by a Student-Newman-Keuls test. Source data are provided as a Source Data file.

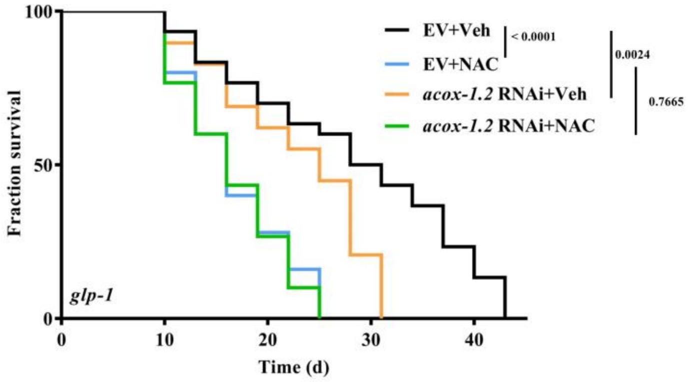

Supplementary Figure 15. H_2O_2 is involved in lifespan extension in *glp-1(e2141ts)* mutants.

Supplementation with N-acetylcysteine (NAC) (5 mM) shortened the lifespan of *glp-1(e2141ts)* mutants. Knockdown of *acox-1.2* by RNAi suppressed the lifespan of *glp-1*(*e2141ts*) mutants, but did not further reduce the lifespan of NAC-treated *glp-1(e2141ts)* mutants. *P*-values were calculated using log-rank test. See survival statistics in Supplementary Data 1. Source data are provided as a Source Data file.

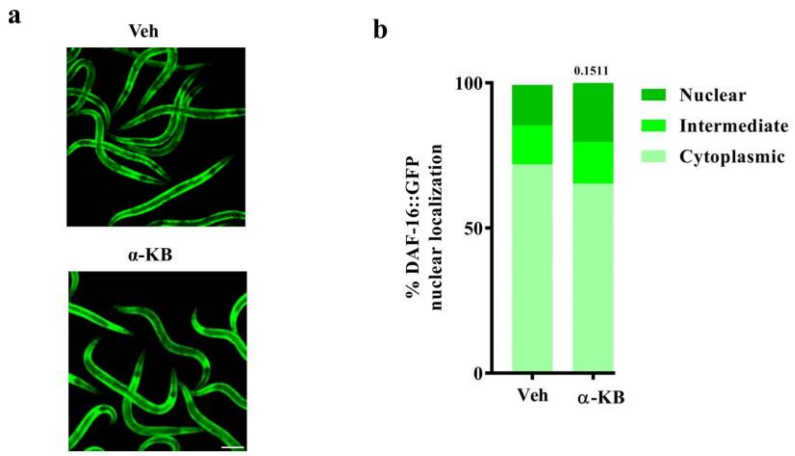

Supplementary Figure 16. Supplementation with α-KB does not induce DAF-16 nuclear localization in worms.

a Representative images of DAF-16::GFP distribution. A similar pattern of distribution was observed in three independent experiments. Scale bars: 100 µm. **b** Quantification of fluorescent intensity in **a**. The percentages of DAF-16 distribution were calculated in total worms (n = 35 worms each experiment). *P*-value was calculated using a Wilcoxon signed rank test. . Source data are provided as a Source Data file.

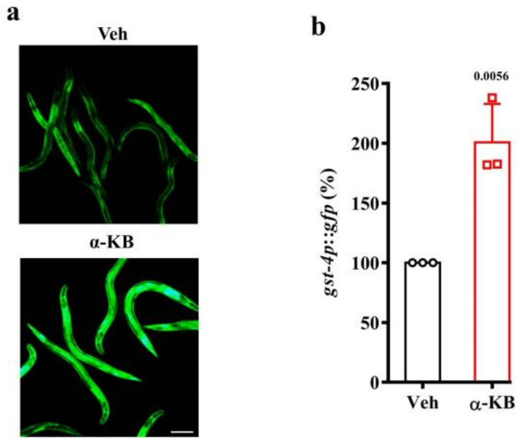

Supplementary Figure 17. Supplementation with α-KB up-regulates the expression of *gst-4p::gfp* in worms.

a Representative images of *gst-4p::gfp* in worms treated with α-KB. A similar pattern of expression was observed in three independent experiments. Scale bars: 100 μm. **b** Quantification of GFP levels in **a**. Supplementation with α-KB increased the expression of *gst-4p::gfp* in WT worms. Data are presented as mean values ± SEM of three independent experiments (n = 35 worms each experiment). *P*-value was calculated using the two sample t-test. Scale bars: 200 μm. Source data are provided as a Source Data file.

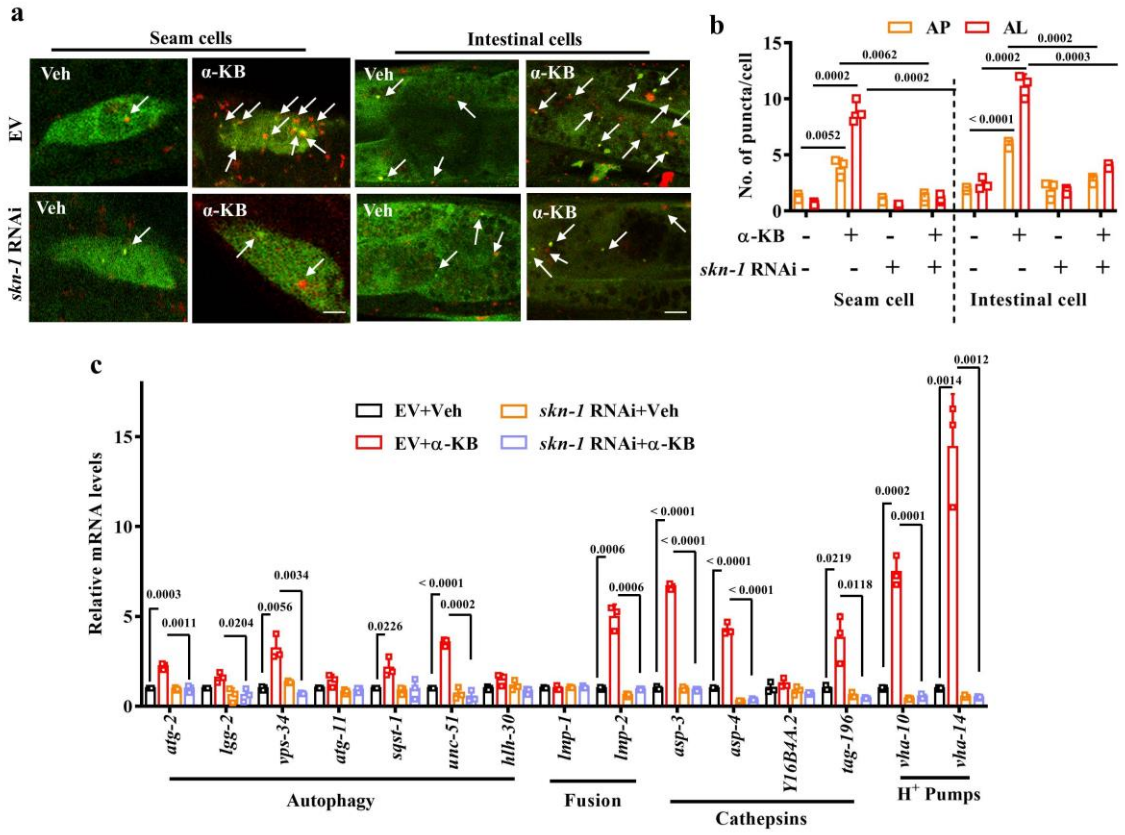

Supplementary Figure 18. SKN-1 is required for α-KB-induced autophagy.

a Representative images of transgenic animals expressing *lgg-1p::mCherry::GFP::lgg-1*. Autophagosomes (AP) are positive for both GFP and mCherry (yellow), whereas autolysosomes (AL) are only positive for mCherry (red). A similar pattern of expression was observed in three independent experiments. Scale bars: 5 μm. **b** Quantification of AP and AL in **a**. Supplementation with α-KB increased the numbers of both AP and AL puncta in worms subjected to empty vector, but not *skn-1* RNAi. Data are presented as mean values ± SEM of three independent experiments (n = 25 worms each experiment). **c** Expressions of autophagy-related genes were measured by qPCR. Most of these genes were up-regulated in WT worms treated with α-KB, which was reduced by knockdown of *skn-1* by RNAi. Data are presented as mean values ± SEM of three independent experiments. *P*-values throughout were calculated using the two sample t-test. Source data are provided as a Source Data file.

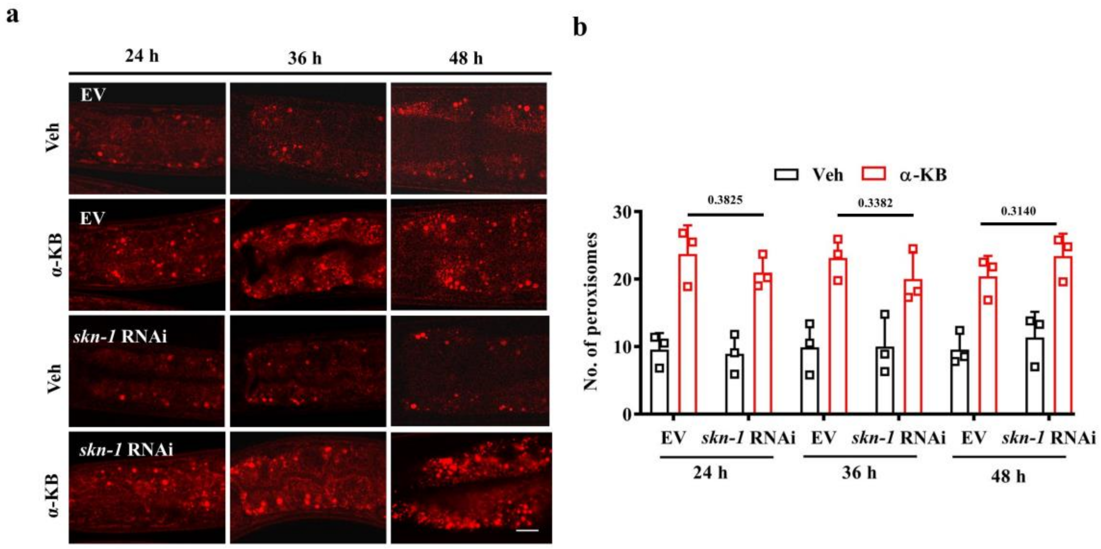

Supplementary Figure 19. The number of peroxisomes in worms treated with α-KB with increasing incubation time.

a Representative images of peroxisomes in worms expressing *vha-6p::mRFP-PTS1*. A similar pattern of expression was observed in three independent experiments. Scale bars: 2.5 μm. **b** The number of peroxisomes was elevated at 24, 36, and 48 h after addition of α-KB. Furthermore, RNAi knockdown of *skn-1* did not affect the increase in the number of peroxisomes in worms after addition of α-KB. Data are presented as mean values ± SEM of three independent experiments (n = 25 worms per experiment). *P*-values were calculated using a one-way ANOVA followed by a Student-Newman-Keuls test. Source data are provided as a Source Data file.

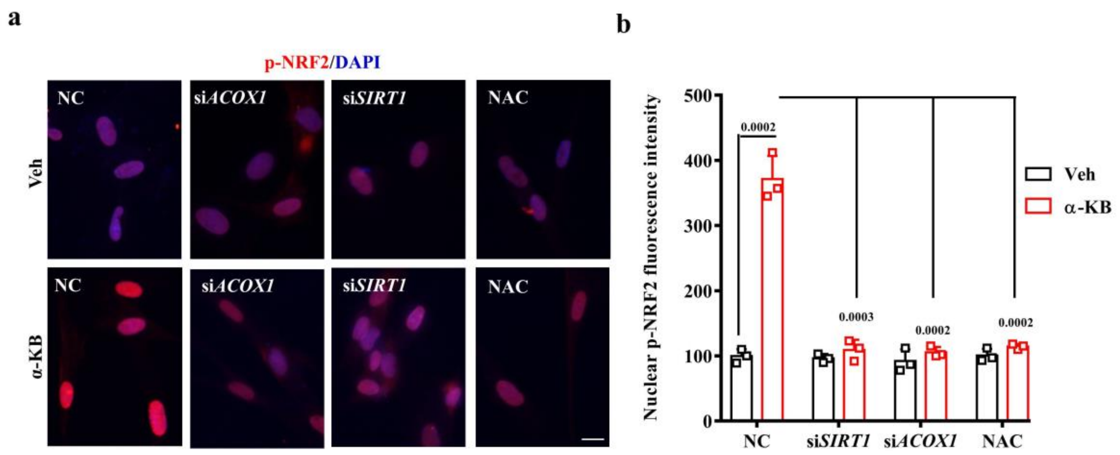

Supplementary Figure 20. Supplementation with α-KB increases the levels of phosphorylated NRF2 in the nucleus.

a Representative images of immunofluorescence staining for anti-phosphorylated NRF2 (Ser40) antibodies. A similar pattern of distribution was observed in three independent experiments. Scale bars: 5 μm. **b** Quantification of fluorescence intensity of phospho-NRF2 in the nucleus. Supplementation with α-KB (500 μM) increased the levels of phosphor-NRF2 in IMR90 cells, which was blocked by RNAi knockdown of *SIRT1*, *ACOX1*, or pre-treatment with NAC (5 mM). *P*-values were calculated using a one-way ANOVA followed by a Student-Newman-Keuls test. Source data are provided as a Source Data file.
