# Supplementary material for: The metabolite alpha-ketobutyrate extends lifespan by promoting peroxisomal function in C. elegans
Source: Nat Commun. 2023 Jan 16;14:240. doi: 10.1038/s41467-023-35899-1 (PMC9842765; doi:10.1038/s41467-023-35899-1)
Supplement: Supplementary file 2 — Description of Additional Supplementary Files [file 41467_2023_35899_MOESM2_ESM.pdf]

Description of Additional Supplementary Files

File name: Supplementary Data 1

Description: Statistical analyses of lifespan experiments.

File name: Supplementary Data 2

Description: List of primers used for qRT-PCR assays.
