# Supplementary material for: The metabolite alpha-ketobutyrate extends lifespan by promoting peroxisomal function in C. elegans
Source: Nat Commun. 2023 Jan 16;14:240. doi: 10.1038/s41467-023-35899-1 (PMC9842765; doi:10.1038/s41467-023-35899-1)
Supplement: Supplementary file 5 — Reporting Summary [file 41467_2023_35899_MOESM5_ESM.pdf]

nature portfolio

Corresponding author(s): Cheng-Gang Zou
Last updated by author(s): Jan 6, 2023

Reporting Summary

Nature Portfolio wishes to improve the reproducibility of the work that we publish. This form provides structure for consistency and transparency in reporting. For further information on Nature Portfolio policies, see our Editorial Policies and the Editorial Policy Checklist.

Statistics

For all statistical analyses, confirm that the following items are present in the figure legend, table legend, main text, or Methods section.

n/a	Confirmed	
☐	☒	The exact sample size (n) for each experimental group/condition, given as a discrete number and unit of measurement
☐	☒	A statement on whether measurements were taken from distinct samples or whether the same sample was measured repeatedly
☐	☒	The statistical test(s) used AND whether they are one- or two-sided<br>*Only common tests should be described solely by name; describe more complex techniques in the Methods section.*
☒	☐	A description of all covariates tested
☒	☐	A description of any assumptions or corrections, such as tests of normality and adjustment for multiple comparisons
☐	☒	A full description of the statistical parameters including central tendency (e.g. means) or other basic estimates (e.g. regression coefficient) AND variation (e.g. standard deviation) or associated estimates of uncertainty (e.g. confidence intervals)
☐	☒	For null hypothesis testing, the test statistic (e.g. F, t, r) with confidence intervals, effect sizes, degrees of freedom and P value noted<br>*Give P values as exact values whenever suitable.*
☒	☐	For Bayesian analysis, information on the choice of priors and Markov chain Monte Carlo settings
☒	☐	For hierarchical and complex designs, identification of the appropriate level for tests and full reporting of outcomes
☒	☐	Estimates of effect sizes (e.g. Cohen's d, Pearson's r), indicating how they were calculated

Our web collection on statistics for biologists contains articles on many of the points above.

Software and code

Policy information about availability of computer code

Data collection	N/A
Data analysis	All statistical analyses were performed on GraphPad Prism 7. ImageJ software was used to analyse fluorescence image.

For manuscripts utilizing custom algorithms or software that are central to the research but not yet described in published literature, software must be made available to editors and reviewers. We strongly encourage code deposition in a community repository (e.g. GitHub). See the Nature Portfolio guidelines for submitting code & software for further information.

Data

Policy information about availability of data

All manuscripts must include a data availability statement. This statement should provide the following information, where applicable:

- Accession codes, unique identifiers, or web links for publicly available datasets
- A description of any restrictions on data availability
- For clinical datasets or third party data, please ensure that the statement adheres to our policy

The data supporting the findings of this study are available within the article and its supplementary materials.

Human research participants

Policy information about studies involving human research participants and Sex and Gender in Research.

Reporting on sex and gender	N/A
Population characteristics	N/A
Recruitment	N/A
Ethics oversight	N/A

Note that full information on the approval of the study protocol must also be provided in the manuscript.

Field-specific reporting

Please select the one below that is the best fit for your research. If you are not sure, read the appropriate sections before making your selection.

☒ Life sciences ☐ Behavioural & social sciences ☐ Ecological, evolutionary & environmental sciences

For a reference copy of the document with all sections, see nature.com/documents/nr-reporting-summary-flat.pdf

Life sciences study design

All studies must disclose on these points even when the disclosure is negative.

Sample size	Sample size was determined accordingly to previous published study and experimental knowledge.
Data exclusions	No data were excluded from the experiments reported.
Replication	Experiments were repeated three time. Experimental findings were reproducible each time.
Randomization	Worms and cells were randomly allocated into different experimental groups.
Blinding	No blinding was applied in the study.

Reporting for specific materials, systems and methods

We require information from authors about some types of materials, experimental systems and methods used in many studies. Here, indicate whether each material, system or method listed is relevant to your study. If you are not sure if a list item applies to your research, read the appropriate section before selecting a response.

Materials & experimental systems

n/a	Involved in the study	
☐	☒	Antibodies
☐	☒	Eukaryotic cell lines
☒	☐	Palaeontology and archaeology
☐	☒	Animals and other organisms
☒	☐	Clinical data
☒	☐	Dual use research of concern

Methods

n/a	Involved in the study	
☒	☐	ChIP-seq
☒	☐	Flow cytometry
☒	☐	MRI-based neuroimaging

Antibodies

Antibodies used	The following antibodies were used for western blot:<br>anti-β-Actin mAb (#sc-8432, 1:4000 dilution, Santa Cruz Biotechnology, Santa Cruz, CA)<br>anti-LC3B(D11) XP Rabbit mAb antibodies (#3868, 1:3000 dilution, Cell Signaling Technology, Danvers, MA);<br>anti-Phospho-AMPKa(Thr172) (40H9) Rabbit mAb (#2535, 1:1000 dilution, Cell Signaling Technology);<br>anti-AMPKa(D5A2) Rabbit mAb antibodies (#2532S, 1:1000 dilution, Cell Signaling Technology);<br>HRP-conjugated anti-mouse (#7074s, 1:3000 dilution, Cell Signaling Technology);<br>HRP-conjugated anti-rabbit (#7076p2, 1:3000 dilution, Cell Signaling Technology).

	The following antibodies were used for immunofluorescence:<br>anti-Nrf2 (CSB-MA614961A0m, 1:500 dilution, Cusabio Technology Llc, Huston, TX);<br>anti-Phospho-Nrf2(Ser40) antibodies (PA5-67520,1:500 dilution, ThermoFisher Scientific);<br>Alexa Fluor 405 anti-mouse IgG(H+L) (A-55057,1:200 dilution, ThermoFisher Scientific);<br>Alexa Fluor 488 anti-Rabbit IgG(H+L) (A-21206,1:200 dilution, ThermoFisher Scientific).
Validation	All antibodies are commercially available. Antibodies employed herein our manuscript were previously reported and routinely used for the application used. The validation and quality control are performed by the corresponding vendors.

Eukaryotic cell lines

Policy information about cell lines and Sex and Gender in Research

Cell line source(s)	The human lung fibroblast IMR-90 cells were obtained from the American Type Culture Collection (ATCC; CCL-186).
Authentication	The cells are not authenticated.
Mycoplasma contamination	The cells are tested for mycoplasma contamination. No mycoplasma contamination is found.
Commonly misidentified lines (See ICLAC register)	No commonly misidentified cell lines were used.

Animals and other research organisms

Policy information about studies involving animals; ARRIVE guidelines recommended for reporting animal research, and Sex and Gender in Research

Laboratory animals	The study involved C. elegans, but no vertebrates, nor higher invertebrates.
Wild animals	The study did not involve wild animals.
Reporting on sex	N/A
Field-collected samples	The study did not involve field-collected samples.
Ethics oversight	No ethical approval or guidance was needed.

Note that full information on the approval of the study protocol must also be provided in the manuscript.
